# Conserved regulation of RNA processing in somatic cell reprogramming

**DOI:** 10.1186/s12864-019-5438-2

**Published:** 2019-01-31

**Authors:** Alexander Kanitz, Afzal Pasha Syed, Keisuke Kaji, Mihaela Zavolan

**Affiliations:** 1 Biozentrum, University of Basel, Basel, Switzerland; 2 RNA Regulatory Networks, Swiss Institute of Bioinformatics, Lausanne, Switzerland; 3 MRC Centre for Regenerative Medicine, University of Edinburgh, Edinburgh, Scotland, UK

**Keywords:** iPS cells, Somatic cell reprogramming, RNA processing, Alternative splicing

## Abstract

**Background:**

Along with the reorganization of epigenetic and transcriptional networks, somatic cell reprogramming brings about numerous changes at the level of RNA processing. These include the expression of specific transcript isoforms and 3’ untranslated regions. A number of studies have uncovered RNA processing factors that modulate the efficiency of the reprogramming process. However, a comprehensive evaluation of the involvement of RNA processing factors in the reprogramming of somatic mammalian cells is lacking.

**Results:**

Here, we used data from a large number of studies carried out in three mammalian species, mouse, chimpanzee and human, to uncover consistent changes in gene expression upon reprogramming of somatic cells. We found that a core set of nine splicing factors have consistent changes across the majority of data sets in all three species. Most striking among these are ESRP1 and ESRP2, which accelerate and enhance the efficiency of somatic cell reprogramming by promoting isoform expression changes associated with mesenchymal-to-epithelial transition. We further identify genes and processes in which splicing changes are observed in both human and mouse.

**Conclusions:**

Our results provide a general resource for gene expression and splicing changes that take place during somatic cell reprogramming. Furthermore, they support the concept that splicing factors with evolutionarily conserved, cell type-specific expression can modulate the efficiency of the process by reinforcing intermediate states resembling the cell types in which these factors are normally expressed.

**Electronic supplementary material:**

The online version of this article (10.1186/s12864-019-5438-2) contains supplementary material, which is available to authorized users.

## Background

Integrated analyses of genomics, epigenomics, transcriptomics, and proteomics data are systematically unravelling the gene regulatory networks underlying the reprogramming of differentiated cells into induced pluripotent stem cells (iPSCs) [1–7], particularly the underlying ‘epigenetic landscape’ [8]. These studies have improved our understanding of the dynamics of cell state transitions and of cell fate decisions, while the vast number of resulting data sets have enabled the development of computational models for predicting regulatory switches, as well as to facilitate cellular reprogramming and transdifferentiation [9, 10].

The ‘molecular roadmap’ of somatic cell reprogramming includes bursts of changes in mRNA, miRNA and histone modification levels during the first three days and at the end of reprogramming. Between these bursts is a less understood period of relatively little transcriptional change. The initial phase resembles a mesenchymal-epithelial transition (MET) [11], a process that takes place during the development of various organs as well as during cancer transformation. Many interactions that have been uncovered in the context of MET or its reversal, epithelial-mesenchymal transition (EMT), are also studied in the context of reprogramming [12]. Interestingly, some of the important changes occur at the level of RNA processing through alternative splicing. For example, the inclusion of 10 variable exons in messages of the adhesion molecule *Cd44*, which is important for MET [13], is driven by ESRP1 and ESRP2 (epithelial splicing regulatory protein 1 and 2) [14]. ESRP1 has recently been reported to also enhance somatic cell reprogramming [15, 16], partly through the alternative splicing of the *Grhl1* (grainyhead like transcription factor 1) [15]. Although evidence of deeply conserved regulators of pluripotency has started to emerge [17], a mammalian pluripotency network that includes splicing regulators has not been reconstructed so far.

Aiming to identify conserved regulators of somatic cell reprogramming we have analyzed mRNA sequencing data obtained in 14 reprogramming studies that were carried out in mouse, chimpanzee and human. We found that functional categories related to ‘RNA processing’ and ‘mRNA splicing’ were strongly over-represented among genes whose expression was higher in iPSCs compared to parental cells, in line with general changes in cell physiology that were reported previously [18–20]. Furthermore, we identified a set of 9 splicing-related genes, which exhibited a highly consistent pattern of expression in iPSCs and parental cells across all species and types of reprogramming methods. Among these, *ESRP1* showed the strongest and most conserved increase in expression in iPSCs compared to parental cells. While the potentiating effect of ESRP1 on reprogramming efficiency had already been reported [15], here we demonstrate that ectopic expression of either *Esrp* paralog accelerates the reprogramming kinetics and increases reprogramming efficiency. Finally, we found that transcripts related to the cytoskeleton, cell adhesion and epigenetic regulation undergo splicing/isoform changes in both human and mouse systems. Our analysis supports the concept that splicing factors with an evolutionarily-conserved cell-type-specificity of expression enforce the identity of the corresponding cell types and can modulate reprogramming efficiency.

## Results

### Expression analysis of somatic cell reprogramming systems across species

One of the first reprogramming studies that employed deep sequencing, investigated somatic copy number mosaicism in 21 iPSC lines derived from the skin fibroblasts of 7 donors [21]. Comparing gene expression of induced pluripotent stem cells (iPSCs) with that of parental fibroblasts in this extensive data set, we found that RNA binding, processing and splicing-related factors were strongly enriched among genes whose expression is increased in iPSCs (Additional file 1). To evaluate the generality of this observation, we have queried the NCBI Sequence Read Archive [22] and identified 14 studies of primate (human, chimpanzee) and rodent (mouse) somatic cell reprogramming (Table 1) that generated deep sequencing data and had replicate samples for both parental cells and reprogramming endpoints (see Methods for details). These studies covered a variety of cell types and reprogramming methods and resulted in a total of 376 Illumina RNA-Seq libraries (Additional file 2; 140 starting and 184 end points as well as 52 intermediate samples, with 4 to 138 samples per study) of varying sequencing depths (Additional file 3: Figure S1A) and read lengths (Additional file 3: Figure S1B).

**Table 1:** Publicly available RNA-Seq data sets analyzed in this study

Id	Accession	Organism	Cell types	Reference
A	SRP011318	Mouse	Adipose progenitor cells, fibroblasts, hematopoietic progenitor cells, iPSCs	[95]
B	SRP016568	Human	Fibroblasts, iPSCs	[21]
C	SRP026281	Mouse	Fibroblasts, iPSCs (chemical)	[96]
D	SRP033561	Mouse	Fibroblasts, iPSCs	[97]
E	SRP033569	Human	Fibroblasts, iPSCs (retrovirus), iPSCs (Sendai virus)	[98]
F	SRP033700	Mouse	Fibroblasts, iPSCs	[99]
G	SRP045688	Mouse	Fibroblasts, iPSCs	[100]
H	SRP045999	Chimpanzee, Human	Fibroblasts, iPSCs, lymphoblastoids (human only)	[101]
I	SRP049340	Human	hiF, hiF-T, iPSCs	[28]
J	SRP052014	Mouse	iPSCs, spermatogonial stem cells	[102]
K	SRP056571	Mouse	Fibroblasts, iPSCs	[103]
L	SRP059670	Mouse	Fibroblasts, iPSCs	[15]
M	SRP063867	Human	Fibroblasts, iPSCs	[104]
N	SRP064357	Mouse	Fibroblasts, iPSCs (chemical)	[105]

The columns indicate NCBI Sequence Read Archive (SRA) accession numbers, the organism or organisms and cell types from which samples were prepared, and a reference to the study for which the data was originally generated

**Fig. 1:**
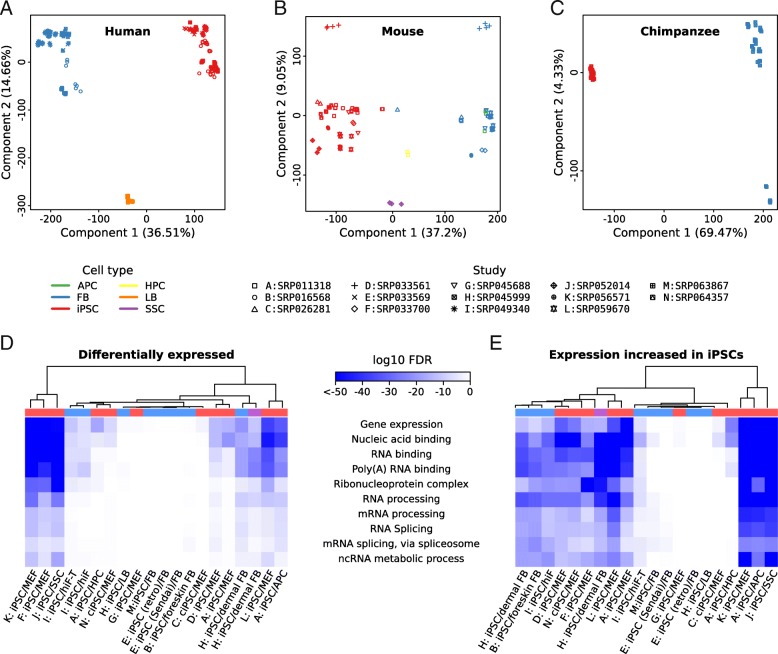
Analysis of gene expression. Principal component analysis of gene expression inferred from human (**a**), mouse (**b**) and chimpanzee (**c**) RNA-Seq libraries. x and y axes correspond to the first and second principal components. Axis labels indicate the amount of variance explained. Colors correspond to cell types (see below for abbreviations), while symbols indicate the individual studies from which the data sets were derived (see also Table 1). (**d** and **e**) Gene set enrichment analyses of genes that were differential gene expression in reprogrammed cells relative to their parental somatic cells. The significance (log10 false discovery rates (FDR) of enrichments in either differentially expressed genes (**d**) or in genes with increased expression (“upregulated”) during iPSC formation (**e**) relative to all expressed genes are shown as heatmaps. GO terms are shown in rows and specific comparisons (data sets) are shown in columns. The organisms from which individual data sets were derived are indicated by the color in the bar above the heatmaps: human - blue; mouse - red; chimpanzee - purple. For clarity, values of log10 FDR were capped at -50. Column dendrograms are based on complete linkage clustering with Euclidean distances. Column (x axis) labels indicate the data sets that were compared, using a 1-letter code that maps to the corresponding Sequence Read Archive accession (see legend above (**d**) and (**e**), right side). Abbreviations are: APC, adipose progenitor cells; ciPSC, chemically induced pluripotent stem cells; FB, fibroblasts; iPSC, induced pluripotent stem cells; hiF, human inducible fibroblast-like cells; HPC, hematopoietic progenitor cells; LB, lymphoblastoid cells; MEF, mouse embryonic fibroblasts; SSC, spermatogonial stem cells. hiF-T cells constitutively express human *TERT* (telomerase reverse transcriptase). See Table 1, Table S2 and the original references for details on specific studies

Principal component analysis of gene expression levels revealed the expected clustering of samples from similar cell types in all organisms (Fig. 1a, b, c). The first principal component explained $$ \raisebox{1ex}{$1$}\!\left/ \!\raisebox{-1ex}{$3$}\right.-\raisebox{1ex}{$2$}\!\left/ \!\raisebox{-1ex}{$3$}\right. $$ of the variance, depending on the species, and clearly separated fibroblasts from iPSCs. Murine adipose progenitor cells (APC) clustered together with fibroblasts, possibly due to their similarity and the fact that protocols to separate these cell types have only recently been established [23]. Human lymphoblastoid (LB) cells, mouse hematopoietic progenitor (HPC) and mouse spermatogonial stem cells (SSC) were located in between fibroblasts and iPSCs in the coordinate system of the first principal component. Thus, batch effects did not mask the relationship between samples, with only the fibroblasts and iPSCs from study SRP033561 being somewhat separated from the bulk of the corresponding cell lines from all other mouse studies along the second principal component (explaining ~9% of the variation). This may be related to the lower mapping rate of reads obtained in the study (Additional file 3: Figure S1C/D).

**Fig. 2:**
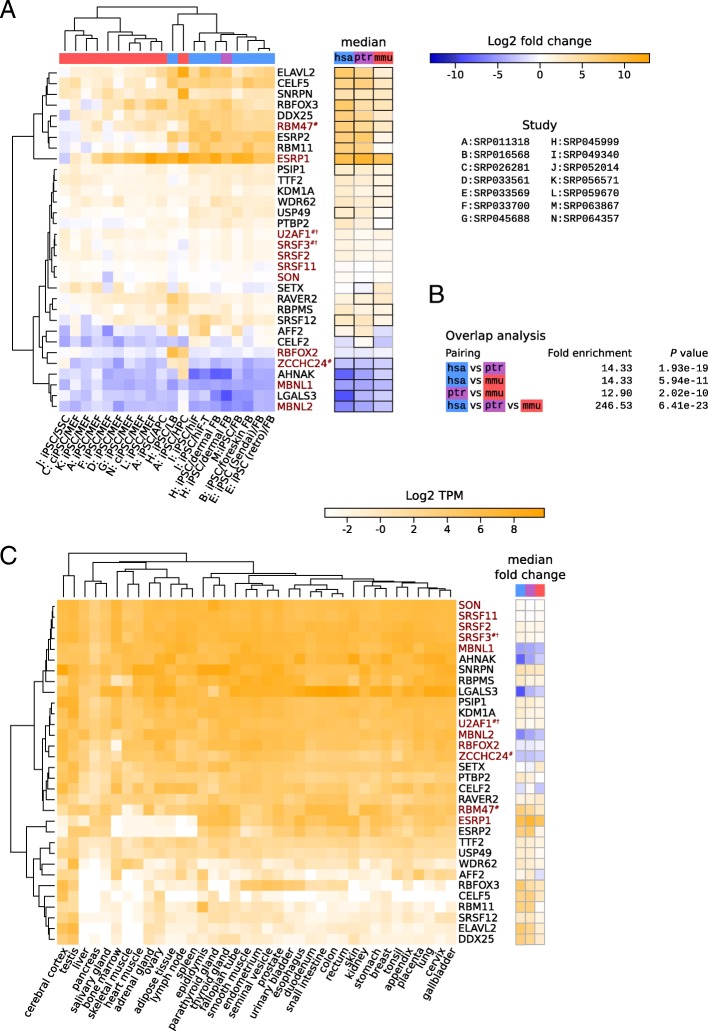
Splicing factors with consistent gene expression changes. (**a**) The left heatmap shows log2 fold-changes in reprogrammed versus parental cells (columns) for genes (rows; human gene symbol) associated with GO term “RNA splicing” (GO:0008380 or descendants). Orange, blue and white indicate higher, lower, or unchanged expression, respectively, in the reprogrammed state. Only genes with (i) a median expression of at least 4 TPM in either endpoint and (ii) a 5-fold difference in expression in at least half of the comparisons of any organism (human, chimpanzee, mouse) are shown. Genes for which one-to-one pairwise orthologous relationships and corresponding gene symbols were unavailable (Ensembl release 84) were disregarded. Putative splicing factors previously implicated in reprogramming (symbols in maroon) [15, 23–27, 66] were included, regardless of them passing the stated criteria. Some of these were not associated with GO:0008380 or descendants(^#^) and/or required manual curation of orthologous relationships(^†^). The right heatmap shows an additional summary: the median fold change for each organism (black borders indicate genes that pass the expression and fold change filters in a given organism). Organism color coding, column labels and dendrograms/clustering as in Fig. 1d/e. See Table 1 and Additional file 2 for details on the used data. (**b**) Evaluation of the significance of overlaps of genes identified in sets of organisms done with the SuperExactTest R package [93]. Fold enrichments and corresponding *P* values are indicated for all 2-way, as well as for the 3-way comparison. (**c**) Expression levels of genes in (A) in a panel of human tissues in The Human Protein Atlas (release 16) [31] (log2 TPM) shown along with the median fold-change observed between iPSCs and parental cells in samples from the three organisms (taken from panel A). Dendrograms and clustering as in Fig. 1d/e

Analysis of gene expression changes in the 20 iPSC-cell of origin comparisons (Table 1, Additional file 4) showed that RNA-binding proteins, RNA processing and splicing factors are enriched among the genes that are *upregulated* in the iPSCs from all three species (Fig. 1d, e, Additional file 5: Figure S2). Their enrichment was detectable but less pronounced among genes that were differentially expressed. This systematic tendency for higher expression in iPSCs is striking, because genes associated with gene ontology terms “RNA splicing” (Additional file 5: Figure S2A/B) and “RNA processing” (Additional file 5: Figure S2C/D) underwent, in fact, smaller changes in expression compared to genes not in these functional categories, as evidenced by shifts of the density functions of log2 fold changes and cumulative distribution functions of absolute log2 fold changes in the expression of genes associated with these terms compared to control genes. Specifically, only ~5% of these genes had absolute log2 fold changes larger than 2 between iPSCs and fibroblasts compared to ~25% of genes from other functional categories, regardless of the study, the protocol used for preparing the samples and the organism from which the cells were derived. Genes in the more general functional category of “gene regulation” did not share the low magnitude of change or the bias towards upregulation (Additional file 5: Figure S2E/F), while genes associated with GO terms “spliceosomal complex” and “ribosome” did (Fig. 2g, h, i and j), indicating a more general change in cell physiology, as reported earlier [18–20].

### Evolutionarily conserved patterns of splicing factor expression in somatic cell reprogramming

We then sought to identify splicing factors with consistent expression changes across studies and species and found that 26 splicing factors exhibited robust and relatively large (median fold change ≥ 5 in studies of at least one species) expression changes (Fig. 2a), 19 having increased and 6 decreased expression in iPSCs compared to fibroblasts. Around a third of splicing-associated genes (9 out of 26) had a median fold change ≥ 5 in all three organisms: *AHNAK* (AHNAK nucleoprotein; also known as desmoyokin), *CELF5* (CUGBP Elav-like family member 5), *ESRP1*, *LGALS3* (galectin 3), *MBNL1/2* (muscleblind like splicing regulator 1 and 2), *RBM47* (RNA binding motif protein 47), *SNRPN* (small nuclear ribonucleoprotein polypeptide N), *ZCCHC24* (zinc finger CCHC-type containing 24). Another six met the expression change cutoff in human and mouse: *ELAVL2* (ELAV like RNA binding protein 2), *RBFOX3* (RNA binding fox-1 homolog 3), *ESRP2* (epithelial splicing regulatory protein 2), *PSIP1* (PC4 and SFRS1 interacting protein 1), *USP49* (ubiquitin specific peptidase 49), *SRSF12* (serine and arginine rich splicing factor 12). This is considerably more than would be expected if expression changes were randomized over genes within individual species (Fig. 2b; *P* value for the overlap across all species <10^-22^; *P* values for pairwise comparisons ranging from ~10^-10^ to ~10^-19^). Only one gene, *AFF2* (AF4/FMR2 family member 2, a gene associated with fragile X-linked mental retardation) increased in expression during the reprogramming of primate cells, but decreased during mouse cell reprogramming. The core set of 9 highly conserved splicing factors included genes coding for recently described regulators of reprogramming such as MBNL1/2 [23], ESRP1, RBM47 and ZCCHC24 [15]. RBFOX2 (RNA binding fox-1 homolog 2), SON (SON DNA binding protein), SRSF2/3/11 (serine and arginine rich splicing factor 2, 3 and 11) and U2AF1 (U2 small nuclear RNA auxiliary factor 1), other splicing factors that have been experimentally linked to the efficiency of reprogramming [24–27], showed considerably less pronounced changes in their mRNA levels in the systems that we analyzed (Fig. 2).

In all species, the gene with the largest and most consistent increase in expression in iPSCs compared to parental cells was *ESRP1* (Fig. 2a). In contrast, its *ESRP2* paralog underwent a similarly large expression change in primate iPSCs, but less pronounced in mouse iPSCs. Time series mRNA sequencing data from human and mouse reprogramming that are also available [15, 28] helped explain this discrepancy. Specifically, we found that while expression of both *ESRP* paralogs steadily and concurrently increased during the reprogramming of human cells (Additional file 6: Figure S3), in mouse cell reprogramming this pattern was shared by *Esrp1*, but not by *Esrp2*. Rather, the expression of *Esrp2* increased only transiently during the reprogramming of mouse cells (Additional file 7: Figure S4). Of note, DDX25, ELAV2, and, to a lesser extent, CELF5 and USP49, exhibited an expression pattern similar to that of ESRP2, with their expression strongly increasing towards the final stage of reprogramming in human, but decreasing in mouse reprogramming systems (Additional file 6: Figure S3/ Additional file 7: Figure S4). RBFOX3, a neuronal marker, and RBM11 showed a similar increase in expression in human reprogramming systems, but their transcripts were almost entirely absent from mouse cells along the entire reprogramming timeline (sum of TPM <1; Additional file 6: Figure S3/ Additional file 7: Figure S4).

Splicing factors whose expression decreased upon reprogramming included *MBNL1/2*, whose siRNA-mediated knock-down was found to increase reprogramming efficiency [23]. Down-regulated genes further encode the canonical splicing factor CELF2, the zinc finger-containing protein ZCCHC24, the neuroblast differentiation-associated scaffolding protein AHNAK that was recently found to bind RNAs [29], and the carbohydrate-binding protein LGALS3 that also functions in splicing [30]. Interestingly, whereas these factors are expressed at high levels across a panel of human tissues [31], the splicing factors whose expression increases upon reprogramming often have a more restricted pattern of expression across human cell types (Fig. 2c). Furthermore, genes whose expression increased most upon reprogramming of primate somatic cells appear to also undergo large expression changes in a large number of cancers (Additional file 8: Figure S5). While *RBFOX3*, *RBM11* (RNA binding motif protein 11) and *DDX25* (DEAD-box helicase 25) are generally downregulated in cancers compared to normal cells, the dysregulation of *CELF5*, *ELAVL2* and *ESRP1* is dependant on the type of cancer. In particular, *ESRP1* expression is heavily upregulated in (endo)cervical cancers (~192-fold), but strongly downregulated (~136-fold) in sarcomas.

When considering genes annotated with the more general “RNA processing” gene ontology term, we identified a total of 53 genes with large and consistent expression changes (median fold change ≥5 across the comparisons of one or more species) between iPSCs and parental cells (Additional file 9: Figure S6A). Similarly to splicing factors, this is much more than expected by chance (Additional file 9: Figure S6B). Beyond the already discussed splicing factors, 9 additional ‘RNA processing’ genes emerged from this analysis, in all three species: *ATXN1* (ataxin 1), *EGFR* (epidermal growth factor receptor), *NR2F1* (nuclear receptor subfamily 2 group F member 1), and *TGFB1* (transforming growth factor beta 1) had lower expression, while *CHD7* (chromodomain helicase DNA binding protein 7), *LIN28A/B* (lin-28 homolog A and B), *MDN1* (midasin AAA ATPase 1), and *TRIM71* (tripartite motif containing 71) had higher expression in pluripotent compared to somatic cells. In particular, the LIN28A protein has been demonstrated to play important roles in reprogramming from very early on, being able to substitute for c-Myc in the “Yamanaka cocktail” [32]. Recently, similar observations have been reported for the LIN28B paralog [33]. Interesting, although these genes are annotated as genes involved in ‘RNA processing’, they are better for other functions, such as DNA or chromatin binding.

### Overexpression of *ESRP*s enhances the reprogramming of mouse embryonic fibroblasts

We were intrigued by the discordant expression pattern of the *ESRP2* paralog in human and mouse reprogramming systems and wondered whether in spite of its transient induction during the reprogramming of mouse cells, this protein can nevertheless increase reprogramming efficiency, as does its paralog. To answer this question we used a previously described reprogramming system of mouse embryonic fibroblasts (MEF) that can be induced to express the set of “MKOS” transcription factors (Myc, myelocytomatosis oncogene; Klf4, Kruppel-like factor 4 (gut); Oct4/Pou5f1, POU domain, class 5, transcription factor 1; Sox2, SRY (sex determining region Y)-box 2) from a genomically-integrated construct. Application of doxycycline to these transgenic MEFs (TNG-MKOS-MEFs) induced the expression of the MKOS factors as well as of mOrange. This allowed the monitoring of MKOS expression during the reprogramming process, while the endogenous Nanog-GFP [34] reporter enabled the detection and quantification of pluripotent stem cell colonies 15 days after MKOS induction.

**Fig. 3:**
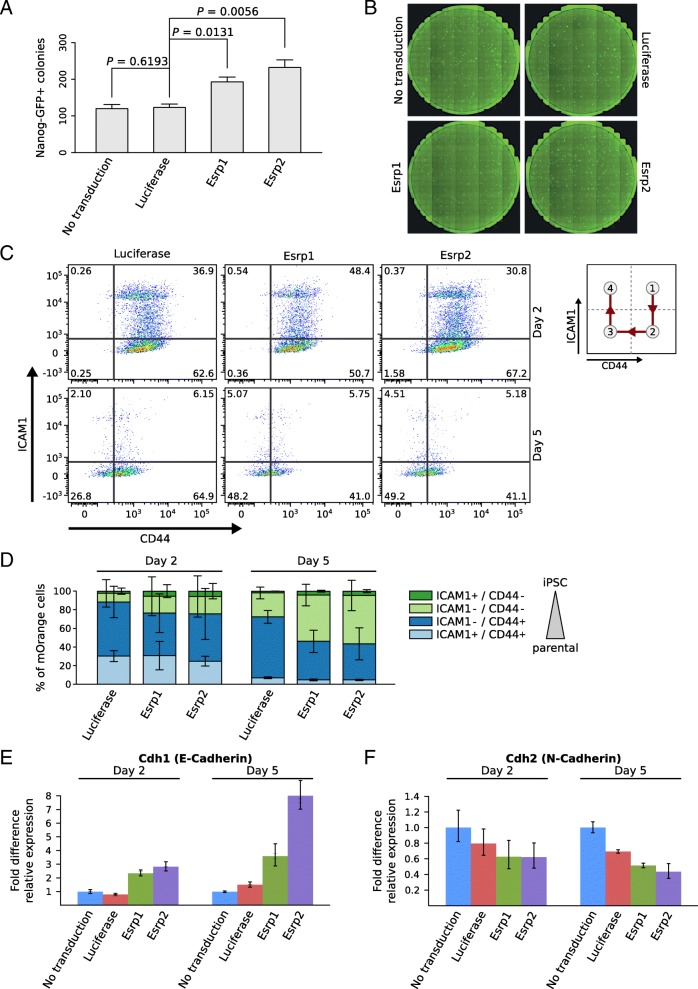
ESRPs enhance reprogramming efficiency of TNG-MKOS-MEFs. (**a**) Number of Nanog-GFP+ colonies counted on day 15 of reprogramming with/without overexpression of either *Esrp1*, *Esrp2* or *Renilla* luciferase expression controls. Error bars show standard deviations and P values are based on two-tailed paired t-tests (n = 3). See Additional file 16 for raw data. (**b**) Representative images of one entire culture dish per condition. Images were taken by a Celigo Imaging cytometer. (**c**) Changes in CD44 and ICAM1 protein levels, measured by flow cytometry during the initial stages of TNG-MKOS-MEF reprogramming (day 2 and day 5). TNG-MKOS-MEFs ectopically expressed either *Renilla* luciferase (left), *Esrp1* (middle) or *Esrp2* (right). The gates define cells in different reprogramming stages as previously described [36]. The expected shift of cells along the reprogramming time course is indicated in the schematic diagram on the right. Data from a representative experiment (of n = 3 experiments) are shown. (**d**) Mean percentages of cells in each gate, with standard deviation computed from the three independent experiments. Gates are labeled ICAM1^+^ / CD44^+^, ICAM^-^ / CD44^+^, ICAM1^-^ / CD44^-^, ICAM1^+^ / CD44^-^ and correspond to the ones in (**c**), clockwise, starting from the top right. An arrow next to the legend indicates the order in which sub-populations appear in a typical reprogramming experiment. See Additional file 16 for raw data. (**e** and **f**) qRT-PCR measurements of epithelial and mesenchymal markers *Cdh1* (**e**) and *Cdh2* (**f**) at day 2 and 5 of the reprogramming process. Samples were normalized to non-transduced samples for each day. *GAPDH* (glyceraldehyde-3-phosphate dehydrogenase) was used as an internal control. Relative fold changes were calculated by the ΔΔCT method [89] . Error bars represent standard deviations (n = 3). See Additional file 16 for raw data

Transduction of TNG-MKOS-MEFs with retroviruses expressing either *Esrp1* or *Esrp2* (Additional file 10: Figure S7A) followed by induction with doxycycline resulted in 1.6 and 1.9 fold more Nanog-GFP positive colonies, respectively, relative to *Renilla* luciferase-transduced or to non-transduced controls (Fig. 3a, and b). Expression of pluripotency markers and their differentiation capacity was confirmed by qRT-PCR, spontaneous differentiation and neuronal lineage differentiation (Additional file 10: Figure S7B-F). Thus, although *Esrp2*’s induction during transcription factor-induced reprogramming of mouse cells is only transient (Additional file 7: Figure S4), it appears to increase reprogramming efficiency to a similar degree as its stably induced *Esrp1* paralog.

### ESRPs enhance MET and accelerate the reprogramming of TNG-MKOS-MEFs

As ESRPs are important for EMT [14] and as the converse MET is an essential stage in MEF reprogramming [35], we investigated the kinetics of the reprogramming process upon expression of either *Esrp* paralog, by monitoring levels of the CD44 and ICAM1 cell surface markers [36]. During reprogramming, mouse fibroblasts convert from CD44^+^ / ICAM1^-^ to CD44^-^ / ICAM1^-^ and then to CD44^-^ / ICAM1^+^ cells, the latter population containing a substantial fraction of pluripotent cells. Flow cytometry-based analysis 5 days after doxycycline induction revealed that the proportion of CD44^-^ / ICAM1^-^ cells was 2-fold higher in *Esrp1/2*-transduced compared to *Renilla* luciferase-transduced TNG-MKOS-MEFs. We also observed a similar trend 2 days after doxycycline induction, but the data is somewhat noisy, likely due to variation in the onset of transgene expression following retroviral transduction. Taken together, these data indicate that both ESRPs accelerate the reprogramming of MEFs (Fig. 3c, d). To further determine whether an accelerated MET underlies the accelerated reprogramming, we measured the expression of epithelial marker *Cdh1* (cadherin 1; E-cadherin) [37] by qRT-PCR. Indeed, we found that *Esrp1/2*-transduced cells expressed 2-fold higher levels of *Cdh1* at day 2 and 3.5-7-fold higher levels at day 5, compared to non-transduced cells (Fig. 3e). The difference in the impact of *Esrp2* and *Esrp1* overexpression on *Cdh1* levels at day 5 is consistent with a previous study demonstrating that the knockdown of *Esrp* paralogs has different effects on *Cdh1* expression in the context of cancer cell motility [38]. Conversely, the expression of the mesenchymal marker *Cdh2* (cadherin 2; N-cadherin) was 1.5 fold lower at day 2 and ~2-fold lower at day 5 in *ESRP1/2*-transduced compared to non-transduced cells (Fig. 3f).

### *Cd44* isoform switching parallels ESRP-induced acceleration of reprogramming

**Fig. 4:**
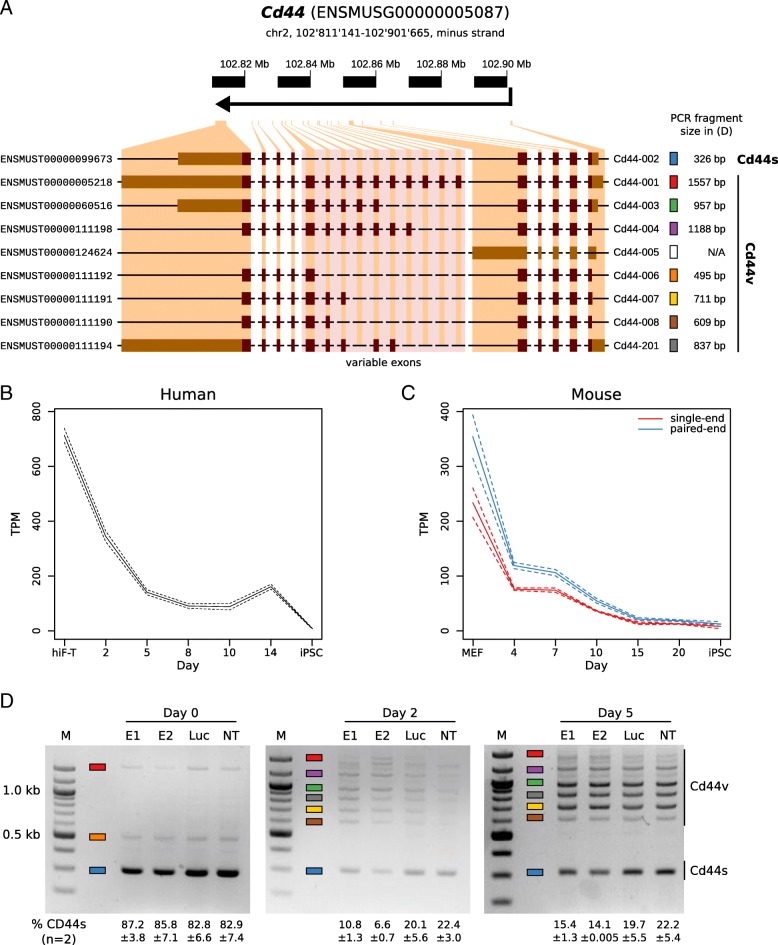
ESRPs regulate alternative splicing of CD44. (**a**) Schematic representation of the murine *Cd44* gene model. Indicated are gene coordinates, chromosome ruler, an arrow indicating the direction of transcription, exons, and transcript isoforms with Ensembl identifiers and symbols. Colored boxes and expected PCR fragment sizes were added next to the transcript symbols to facilitate interpretation of (D). The mesenchymal or “standard” (Cd44s) and the epithelial or “variable” (Cd44v) isoforms are highlighted. (**b** and **c**) Gene expression profile of the *Cd44* gene in human (**b**; study SRP049340) [28] and mouse (**c**; study SRP059670) [15] reprogramming time series (x axes). Expression levels (y axes) are given in transcripts per million (TPM). Single- (red) and paired-end (blue) RNA-Seq libraries from study SRP059670 were analyzed separately. Dashed lines indicate 95% confidence intervals. (**d**) Semi-quantitative RT-PCR of *Cd44* isoforms at various time points (days 0, 2 and 5) of TNG-MKOS-MEF reprogramming. Cells ectopically expressed either *Esrp1* (E1), *Esrp2* (E2), *Renilla* luciferase (Luc), or no transgene (not transduced, NT). M, 100 bp DNA marker (NEB, #N3231S). The colored boxes represent the different transcript isoforms as defined in (**a**) Band intensities for the Cd44s isoform, as quantified by AzureSpot (Azure Biosystems), are indicated below the gel photos (n = 2) as in (**d**). See Additional file 16 for raw quantification data for all bands

The key role of ESRPs in EMT is partly due to them promoting the inclusion of variant exons 6-15 in *Cd44* transcripts, leading to a switch from the ‘standard’ (“*Cd44s*”) isoform to epithelial (“*Cd44v*”) isoforms [39] (Fig. 4a). As mentioned above, *Cd44* gene expression is down-regulated during reprogramming, both at the protein [36] and at the mRNA level (Fig. 4b, and c). In addition, using equal amounts of *Cd44* cDNA as starting material, we found by semi-quantitative RT-PCR that the relative abundance of *Cd44v* isoforms was higher in cells transduced with *Esrp1/2* (Fig. 4d) compared to non-transduced cells. These results demonstrate that similar to ESRP1, ESRP2 increases the efficiency of somatic cell reprogramming in mouse, accelerating the induction of MET and the overall kinetics of the process.

### A network of conserved splicing changes in human and mouse somatic cell reprogramming

Splicing patterns are known to change relatively rapidly during mammalian evolution [40]. Nevertheless, given the strong conservation of the splicing regulators, we sought to identify genes and pathways in which reprogramming-related splicing events occur in both human and mouse. To evaluate the significance of alternative splicing changes between reprogramming starting and end points, we quantified the inclusion of annotated alternatively spliced exons in terms of ‘percent spliced in’ (PSI) values, a measure indicating the fraction of transcripts that are consistent with a specific splicing event among all transcripts generated from the corresponding gene, across all samples. Principal component analysis of PSI values of all quantified events again revealed the expected clustering of the samples by cell type (Additional file 11: Figure S8A/B), although the first two principal components explained only 15-18% of the variance. Gene ontology analysis of genes that underwent significant (*P* ≤ 0.05) splicing changes showed relatively little overall conservation across species (Additional file 11: Figure S8C).

**Fig. 5:**
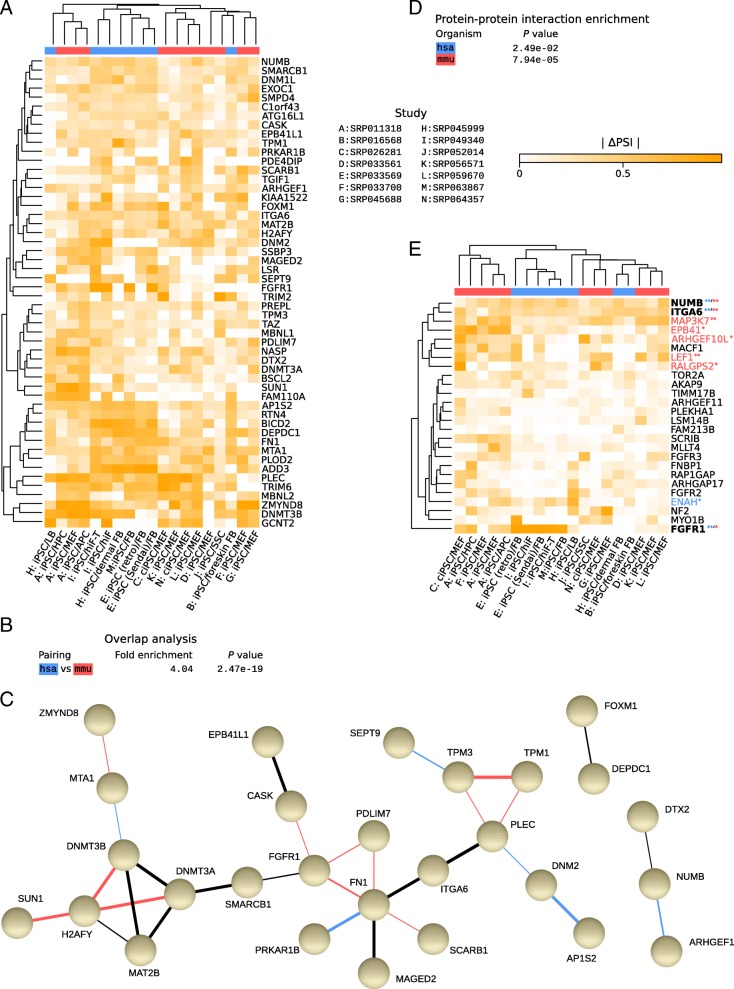
Consistent changes in alternative splicing. (**a**) Heatmap indicating absolute changes in ‘percent spliced in’ (ΔPSI) of splice variants (rows) between reprogrammed and parental cells (columns). Gene symbols (human symbols shown) indicate the genes that harbor any of the 500 most robust AS events, ranked by absolute mean z-scores across comparisons, in both human and mouse, with the shown absolute ΔPSI values corresponding to the most robust event for a particular gene. Only events with a minimum combined expression of ≥2 TPM for all transcripts involved in the event (median expression across all corresponding samples) were considered. Genes for which one-to-one pairwise orthologous relationships and corresponding gene symbols were unavailable in Ensembl release 84 were disregarded. Column labels, dendrograms/clustering and color coding of species as in Fig. 1d/e. (**b**) Overlap enrichment analysis of genes in (**a**) computed and represented as in Fig. 2b. (**c**) Combined interaction network of proteins corresponding to the genes in panel (**a**), derived from STRING [42]. STRING analyses were carried out individually for human and mouse, and the results were merged based on orthology relationships. The widths of connecting lines indicate STRING confidence levels. Interactions reported for both organisms are shown in black, those annotated for human or mouse only are shown in blue and red, respectively. Proteins with no interacting partners within the set were omitted. (**d**) STRING protein-protein interaction enrichment *P* values. (**e**) As in (**a**), but only for reported ESRP targets [15, 39] and corresponding AS events. Here, events were not filtered based on expression. Genes with symbols in blue, red or bold harbor the most robustly differentially spliced events (among top 100 or 500 AS events of a given organism, as indicated by one or two correspondingly colored asterisks, respectively) in human, mouse, or both organisms, respectively

We then extracted in each species the 500 splicing events with the highest mean z-score across reprogramming data (we have included the top 100 events in each species for reference: Additional file 12: Figure S9 and Additional file 13), identified the genes from which they originated and then the orthologs in human and mouse. We found 52 genes that are conserved targets of alternative splicing during reprogramming (Fig. 5a), which is 4-fold more than expected by chance (Fig. 5b; *P* value of multi-set intersections = 2.47e-19). Thus, in spite of relatively little conservation of splicing across species [41], a substantial number of genes undergo splicing changes during the reprogramming of both human and mouse somatic cells. The majority of these genes are connected through protein-protein interactions according to the STRING analysis tool [42] (Fig. 5c), which is again more than expected by chance (Fig. 5d; *P* values of protein-protein interaction enrichment of 0.025 for human and 7.94 x 10^-5^ for mouse). The largest clusters of conserved splicing targets correspond to epigenetic regulators, cell adhesion and cytoskeleton-associated molecules, known modulators of cell fate (Fig. 5c). Focusing on genes whose splicing is regulated by ESRP1/2 [15, 39], we found that only few undergo significant splicing changes in both human and mouse (Fig. 5e). However, those that do, i.e. *NUMB* (NUMB, endocytic adaptor protein), *ITGA6* (integrin subunit alpha 6) and *FGFR1* (fibroblast growth factor receptor 1), are part of conserved networks linked to pluripotency (Fig. 5c).

We have further conducted a reprogramming time course experiment in the system described in the previous sections, focusing on the early stages of the process (days 0-5 following doxycycline induction). A time point representing iPSCs (day 15) was also included. We have then selected those of the top 100 differentially spliced events identified for mouse (see above) that correspond to genes from included in the described network of conserved splicing changes (Fig. 5c) or predicted to be Esrp targets (Fig. 5e). Plotting the corresponding percent spliced in (PSI) values for each day and condition (overexpression of *Esrp1*, *Esrp2* or Luciferase following retroviral transduction or no transduction) largely verified differential splicing of these events between TNG-MKOS-MEFs and iPSCs (Additional file 14: Figure S10A). However, although the introduction of *Esrp1* or *Esrp2* cDNAs demonstrably led to a considerable and specific increase in their respective gene expression levels in the early stages of reprogramming (up to day 4; Additional file 14: Figure S10B), we did not identify splicing patterns that were specific to their overexpression compared to control treatments (Additional file 14: Figure S10A). We have also specifically looked at the expression of *Grhl1* isoforms, as *Grhl1* splicing has previously been reported to be affected by Esrp1 during reprogramming (Cieply et al. 2016). These isoforms were not included in the SUPPA index of splicing events because the short *Grhl1* isoform lacks multiple exons, and these complex splicing patterns are not analyzed by the SUPPA software. While both *Grhl1* isoforms are largely absent in TNG-MKOS-MEFs (Additional file 14: Figure S10C; day 0) and only expressed at low levels in iPSCs (day 15), their expression steadily increases between days 1 and 5, reaching peak abundances of approximately TPM = 5 (Grhl1-201) and TPM = 8 (Grhl1-202). However, while the relative abundance of the isoforms (Additional file 14: Figure S10D; fraction of each isoform by total gene expression) appears to be changing between days 2, 5 and 15, a clear influence of elevated *Esrp1* or *Esrp2* levels could not be observed (Additional file 14: Figure S10D). One possible reason for the lack of a consistent effect of *Esrp* overexpression on the splicing of *Grhl1* (Additional file 14: Figure S10D) and other genes (Additional file 14: Figure S10A) might be the technical limitations of accurate quantification of isoforms, particularly of those with low abundances, leading to inaccurate PSI estimations [43]. However, it may also be possible that Esrp-induced changes in splicing patterns of the studied events are saturated at endogenous levels or affect splicing at later time points.

## Discussion

Stem cells have long held great promise for regenerative medicine [44]. Thus, the demonstration that the gene regulatory program of fully differentiated mammalian cells can be overridden by temporarily expressing pluripotent stem cell-specific transcription factors to generate induced pluripotent stem cells (iPSCs) [45, 46] had a profound impact on basic and medical research [47]. iPSCs are used as experimental models to study a wide range of diseases and to discover relevant drugs via chemical library screenings [48]. They also serve as a basis for grafting organoids and tissues [49–51]. A first clinical trial involving patient-derived iPSCs is currently ongoing [52] and several more will be launched within the next decade [53]. Despite this tremendous progress, our understanding of the process is still limited [54] and the efficiency of transcription factor-driven reprogramming remains in the percentage range [55]. Approaches to increase it are in high demand.

Many different types of modulators have been already identified. Very potent is vitamin C, which substantially increases the efficiency of iPSC colony formation [56] by promoting epigenetic remodeling through histone and DNA demethylation [57, 58]. MicroRNAs of the embryonically-expressed miR-302/367 cluster also increase the efficiency of transcription factor-induced reprogramming, repressing targets that are involved in the cell cycle, chromatin remodeling, vesicular transport and epithelial-to-mesenchymal transition [59]. The related miRNAs of the mouse-specific miR-290-295 cluster have been reported to contribute to the stabilization of the stem cell state by targeting epigenetic regulators [60–63].

Following discoveries that core transcriptional drivers of reprogramming such as OCT4/POU5F1 and NANOG (Nanog homeobox) are regulated by alternative splicing [27, 64, 65], splicing factors have also been added to the repertoire of modulators of the reprogramming process. The general splicing factor SRSF2 has been linked to the pluripotency as it affects the balance of *MDB2* (methyl-CpG binding domain protein 2) isoforms, which are part of a complex pluripotency circuit involving the OCT4/POU5F1 transcription factor and the miR-302/367 miRNAs [27]. Conversely, SRSF11 acts as a roadblock for reprogramming, its RNAi-mediated knockdown leading to the rapid emergence of pluripotency-specific isoforms [66]. MBNL1/2-dependent alternative splicing results in a change in the sequence-specificity of the FOXP1 transcription factor, with consequences for the efficiency of reprogramming [23, 67]. MBNL1 and MBNL2 are deeply conserved pluripotency regulators, in functional antagonism with CELF factors in the stem cells of planarian flatworms [17].

Many splicing factors that have so far been found to modulate reprogramming efficiency act as reprogramming roadblocks [12]. This may be because many studies used small RNA-based screening to deplete individual factors in early stages of reprogramming. However, as we have found here, most splicing regulators increase in expression during the reprogramming time course, and one may expect them to rather promote reprogramming. Indeed, this was demonstrated for *Esrp1* [15, 16], the splicing factor most strongly upregulated in iPSCs relative to parental cells (Fig. 2a).

While conventional human iPSCs and mouse iPSCs represent ‘primed’ and ‘naive’ pluripotent states, respectively, requiring distinct signaling pathways for their self-renewal [68], the differences between these states and the species-specific aspects of reprogramming are not fully understood. Our analysis revealed that among the core splicing regulators, *ESRP2* has a different expression dynamics in mouse compared to human, mirroring the monotonic increase in expression of its *ESRP1* paralog in human reprogramming systems, but undergoing only transient induction in mouse (Additional file 7: Figure S4). Nevertheless, we found that just as ESRP1, ESRP2 also accelerated the kinetics and increased the efficiency of mouse cell reprogramming (Fig. 3a, b, c and d).

Although splicing networks appear to evolve relatively fast and splicing events are not strongly conserved in evolution [40], we identified a core set of conserved splicing targets that underwent significant changes across a large number of reprogramming systems. This set is likely incomplete, as the human and mouse transcriptomes that we used for our analysis of isoform usage are still not fully annotated. Furthermore, methods to quantify isoform usage are still in development, and the quantifications are not very accurate, particularly for low abundance transcripts [43]. Nevertheless, we identified conserved splicing targets in excess of what is expected by chance. They encode proteins that promote remodelling at the cellular and chromatin level, and are themselves subject to many regulatory influences. *Cd44* is one example: although the gene is strongly down-regulated at the level of transcription in the early stages of reprogramming, splicing of the produced transcripts also changes, leading to isoforms that contain epithelium-specific variable exons (*CD44v*) and are expressed at low levels. Also heavily regulated are factors that deposit or read DNA methylation marks, such as *DNTM3B* (DNA methyltransferase 3 beta): apart from undergoing alternative splicing, its expression is further modulated by embryonically-expressed miR-290-295 miRNAs as well as MECP2 (methyl CpG binding protein 2), which is in turn regulated by the related family of miR-302/367 miRNAs [63]. Isoform switching and miRNA-dependent regulation has been reported for other methyl-CpG binding domain proteins as well [27]. Multiple layers of regulation probably ensure that the activity of these epigenetic regulators is precisely controlled.

## Conclusions

Through a comprehensive analysis of RNA sequencing data sets obtained in studies of mouse, chimpanzee and human somatic cell reprogramming, we uncovered an extensive set of splicing factors that undergo conserved changes in expression. In contrast to *ESRP1*, which undergoes strong upregulation across all reprogramming studies, the pattern of expression of its paralog, *ESRP2*, differs between species. Nevertheless, both of these proteins accelerate the kinetics and increase the reprogramming efficiency of MEFs. The RNA sequencing data further allowed us to reconstruct networks of splicing targets that are conserved between species. They correspond to proteins involved in subcellular structure and traffic as well as in DNA modification. Although transcription factors have been instrumental in changing cell fates, the efficiency of somatic cell reprogramming remains limited. A variety of molecules ranging from small metabolites and miRNAs to splicing regulatory proteins have been found to modulate the process. An improved understanding of these factors’ functions will enable a more controlled and efficient engineering of cell identity.

## Methods

### Gene and protein nomenclature

Gene symbols in this manuscript are italicized when referring to the gene itself, as well as derived transcripts and cDNAs (e.g. the *ESRP1* isoforms), but not when the corresponding proteins are referenced (e.g. the ESRP1 protein). Only when unambiguously referring to mouse genes, cDNAs and mRNAs, symbols are specified in title case (e.g. *Esrp1*). In all other cases, including in cases where general statements are made about genes that are conserved across primates and rodents, symbols are denoted in all capitals (e.g. *ESRP1* for the gene and ESRP1 for the protein). When a gene is first referenced, the corresponding human name/description is specified in parentheses right after the symbol. In some cases, popular symbols or names are indicated in addition to the (latest) official symbols/names.

### RNA-Seq study search and selection

To obtain previously released data on iPSC reprogramming and differentiation, the Gene Expression Omnibus [69] was queried for relevant keywords ('iPSC' OR 'iPSCs' OR 'iPS cells'; 'somatic' AND 'reprogramming'; 'induced' AND 'reprogramming') on September 14th, 2016. Moreover, to be able to carry out the downstream analyses, we required that the data were derived from organisms whose genome sequence and corresponding gene annotations are available at Ensembl [22, 70]. We inspected the corresponding studies that had Illumina RNA-Seq-based sequencing data deposited at the NCBI Sequence Read Archive [22] and retained only those studies that include at least two replicates for each of iPSCs and the corresponding tissues or cell lines of origin. The Illumina RNA-Seq data of all included studies were downloaded from the NCBI Sequence Read Archive and converted to FASTQ format using the SRA Toolkit [71].

### Genomes and other public resources

Genome sequences, gene annotations and sequences of mature mRNAs for human (GRCh38), mouse (GRCm38) and chimpanzee (CHIMP2.1.4) were obtained from Ensembl [70], release 84. On the genome level, unassembled regions, haplotype and patch regions were disregarded. Genes annotated on mitochondrial DNA and regions not assembled into chromosomes were dropped from the gene annotations and only genes of the following biotypes were kept: 'protein_coding', 'lincRNA', 'processed_transcript', 'antisense'. Human and mouse transcripts were further filtered according to their transcript support level (unavailable for chimpanzee transcript annotations), with only transcripts of support levels 1 through 3 being retained. This amounts to the minimal requirement that the transcript is supported by a single non-suspect EST. For analyses of tissue-dependent gene expression, we used the data (in transcripts per million; TPM, in a each sample, log2-transformed) from The Human Protein Atlas (release 16) [31]. Log-transformed expression data for select genes in cancer and corresponding normal tissue were obtained from The Cancer Genome Atlas [72] through the FireBrowse ‘mRNASeq samples API’ [73]. Log2 fold changes for available tumor versus normal tissue comparisons were computed based on the obtained median expression values.

### RNA-Seq analysis

Genome sequences, transcript sequences and gene annotations were indexed using STAR v2.4.1c [74], kallisto v0.42.3 [75] and SUPPA v2.1 [76] for read-to-genome mapping, estimation of transcript abundance and quantification of exon inclusion, respectively. Gene annotations were provided during STAR index generation. Cutadapt v1.8.3 [77] was used to remove poly(A) tail fragments from reads of all sequencing libraries. Sequenced reads, in FASTQ format, were aligned to the genome with the STAR aligner (Figure S1C/D for mapping rates). Transcript abundances were estimated with kallisto, and based on these, the relative usage of transcript isoforms and alternative splicing events was quantified with SUPPA. The means and standard deviations of the fragment length distributions required by kallisto for estimating transcript abundances from single-end sequencing libraries were set to 300±100 for single-end libraries obtained from the NCBI Sequence Read Archive. The indexing of genome resources and processing of RNA-Seq samples was performed with the help of the Anduril workflow framework v1.2.23 [78]. Estimates of gene expression were obtained by summing transcript abundances and raw read counts (from kallisto; see data sets 1-6 in [79]) for transcripts and reads corresponding to individual genes. Before calculating principal components, the gene-by-sample gene expression matrix (in transcripts per million, TPM) for a given organism was first log2-transformed and then zero-centered by columns and rows.

### Differential expression and splicing analyses

To compare gene expression between sample types of interest (e.g. reprogrammed versus differentiated cells from a given study), gene-level read count estimates [80] for the corresponding samples (Additional file 2) were used as input to the R/Bioconductor package edgeR v3.12.0 [81] for differential gene expression analysis (see data sets 7-12 in [79] for comprehensive summaries of log fold changes and false discovery rates). Unless mentioned otherwise, genes with a false discovery rate (Benjamini-Hochberg method) of less than 0.05 were considered differentially expressed. Gene set enrichment analyses were performed with Ontologizer 2.1 (Build: 20160628-1269) [82] and calculation method “Term-For-Term”. The ontology file (downloaded on 2016-12-24; in OBO 1.2 format) was obtained from The Gene Ontology Consortium [83]. Genes of a given organism associated with each GO term were obtained from Ensembl BioMart [84] Archive March 2016 version (corresponding to Ensembl release 84) and converted to the GAF 2.0 format by a custom script. ‘Percent spliced in’ (PSI) measures calculated with SUPPA for each annotated alternative splicing event (see data sets 13 and 14 in [79] for PSI values in human and mouse, respectively) were further supplied to SUPPA's diffSplice function [85] to identify differentially spliced events in comparisons of interest (see data sets 15-18 in [79] for comprehensive summaries of the resulting ΔPSI and associated *P* values).

### Orthologous genes & conservation analyses

Orthologous genes were obtained from Ensembl BioMart [84] (Archive March 2016 version, corresponding to Ensembl release 84) and filtered for one-to-one relationships. Using the resulting table of corresponding Ensembl identifiers for human, mouse and chimpanzee (differential gene expression analysis only) orthologs, as well as the Ensembl gene annotations (release 84, see above) for individual species, Ensembl identifiers were converted to human gene symbols. Only those genes were kept whose Ensembl identifier could be unambiguously matched to a unique gene symbol.

### Reproducing the computational analyses

Generally, the indicated tools were ran according to their primary use cases, thus requiring no or only very little modification of default values. In other words: if at all, default values Exhaustive information on how computational analyses were performed and instructions for replicating the analyses are available on GitHub [86].

### Cell culture

Murine transgenic Nanog-GFP embryonic stem cells were generated from the E14Tg2a mouse ES cell line in the Smith lab as described previously (TNG-ESCs) [34] and donated to the Kaji lab. TNG-MKOS-ESCs were derived from TNG-ESCs by gene targeting the Sp3 locus with a vector containing a 2A peptide-linked "Yamanaka factors" (MKOS) gene cassette followed by ires-mOrange under a tetracycline inducible promoter and a reverse tetracycline transactivator (rtTA) under a constitutive CAG promoter, as described previously [87]. Chimeric mouse embryos were generated with TNG-MKOS-ESCs via morula aggregation. TNG-MKOS-MEFs and wild-type MEFs were isolated at E12.5 from chimeric and wild-type embryos, respectively, as described previously [36]. NMuMG mouse mammary gland epithelial cells [88] and Phoenix-ECO cells were obtained from the American Type Culture Collection (ATCC; numbers CRL-1636 and CRL-3214, respectively).

Phoenix-ECO cells and TNG-MKOS-ESCs were cultured in Glasgow Minimum Essential Medium (GMEM; Sigma Aldrich, G5154), supplemented with 10% fetal bovine serum (Invitrogen, 10270-106, Lot 41A1520K), 1X non-essential amino acids (100X, Invitrogen, 11140-036), 1X Pen/Strep antibiotics, 1 mM sodium pyruvate (Invitrogen, 15140-122), 0.1 mM 2-mercaptoethanol (Life Technologies, 31350010), 2mM L-glutamine (Invitrogen, 25030-024) and 100,000U/ml of leukemia inhibitory factor (LIF) (from Kaji lab, SCRM, University of Edinburgh). This medium hereafter is referred to as normal medium. Normal medium was supplemented with FGF-2 (5 ng/ml, Preprotec, 100-18B) and heparin (1 μg/ml, Sigma Aldrich, H3149) to culture wild-type and transgenic MEFs (TNG-MKOS-MEFs). Reprogramming medium consisted of normal medium with 1 μg/ml doxycycline (Sigma Aldrich, D9891-1G), 10 μg/ml vitamin C (Sigma Aldrich, 1000731348) and 500 nM Alk5 inhibitor (A83-01, Tocris Bioscience, 2939). NMuMG cells were cultured in Dulbecco's modified Eagle's medium (DMEM; Sigma Aldrich, D5671) with high glucose and L-glutamine, supplemented with 10% fetal bovine serum (Sigma-Aldrich, F7524).

### Retroviral vectors and transductions

Retroviral vectors were constructed via an attR1/R2 Gateway cloning cassette (Invitrogen). *Esrp1* and *Esrp2* cDNAs were PCR-amplified from NmuMG cells; *Renilla* luciferase cDNA was PCR amplified from psiCHECK-2 (Promega AG, C802A). See Additional file 5 for a list of the used primers. cDNAs were cloned into the pENTR2B Gateway entry vector (Invitrogen, A10463) using *Eco*RI sites. Mutation-free inserts were gateway-cloned into a pMXs retro-vector [45] using LR clonase (Invitrogen, 11791-020). Pseudo-retroviral particles were produced by transfecting 10 μg of pMXs retro-vectors expressing transgenes, separately for 48 hrs in a 100 mm plate seeded with 2.2x10^6^ Phoenix-ECO cells (ATCC, CRL3214). Medium collected after 48 hrs of transfection was filtered with 0.45 micron filter and mixed with polybrene (TR-1003-G, Millipore) for transductions.

### Reprogramming of transgenic mouse embryonic fibroblasts

TNG-MKOS-MEFs were isolated from 12.5 dpc chimeric embryos and contribution levels of transgenic cells were measured by mOrange expression upon doxycycline administration. For all reprogramming experiments conducted in this study, 3% TNG-MKOS-MEFs were seeded with 97% of wild-type MEFs in a gelatinized 6 well culture plate. A total of 1x10^5^ cells (3000 TNG-MKOS-MEFs and 97000 WT-MEFs) was seeded in each well of a 6 well plate. Each well was transduced with 2 ml of retroviral particles expressing *Esrp1*, *Esrp2* and *Renilla* luciferase for 4 hours with polybrene (1ug/ml, Millipore, TR-10003-G). After 4 hours medium was replaced with reprogramming medium to induce reprogramming of TNG-MKOS-MEFs. Expression of the transgenes was measured by qRT-PCR after 4 days of transduction and induction of the reprogramming experiment (Figure S7A). Colony counting was done after 15 days of reprogramming induction. The number of colonies was determined by manual counting and validated with the Celigo software.

### Quantitative real-time PCR and RT-PCR

For qRT-PCRs assays, total RNA was extracted using TRI reagent (Sigma Aldrich, T9424) and subjected to DNase digestion with RQ1 DNase (Promega, M6101) followed by phenol-chloroform (Sigma Aldrich, P3803) purification. 1μg of total RNA was used for cDNA synthesis using SuperScript III (Invitrogen, 18080-044) reverse transcriptase according to the manufacturer’s protocol. 10 ng of cDNA per sample was added to Power SYBR Green PCR Master Mix (Applied Biosystems). Assays were performed in triplicates. See Additional file 15 for a list of the used assays and primers. Unless specified otherwise, *GAPDH* levels were used as internal control, and the samples from non-transduced controls or MKOS-ESCs [87] were used as external controls. Relative fold changes were calculated according to the ΔΔCT method [89]. RT-PCR products were stained with RedSafe (iNtRON Biotechnology) and run on a 1.5% agarose gel. DNA was visualized at a wavelength of 290 nm. For the *Cd44* RT-PCR assays, a *Cd44* isoform (ENSMUST00000005218) was PCR amplified (see Additional file 15 for the primer sequences) and purified by the QIAEX II gel extraction kit (Qiagen, 20021). The DNA concentration was quantified by spectrophotometry (NanoDrop). A series of 10-fold dilutions of this DNA was used as a template for the generation of a standard curve in which CT values were plotted against the concentration of the template. The amounts of cDNA to use in order to obtain equal amounts of *Cd44* in each RT-PCR assay were calculated by linear regression from the standard curve. Pixel intensities of each band of the resulting gel photos were quantified by the AzureSpot analysis software (Azure Biosystems).

### Flow cytometry, cell sorting and time course mRNA-sequencing

Double staining of CD44 and ICAM1 was performed as previously described [36, 88]. Briefly, TNG-MKOS-MEFs infected with retroviral particles and treated with doxycycline to induce reprogramming as described above, were trypsinized, filtered through a cell strainer and stained with fluorophore-conjugated antibodies of ICAM1-Biotin (eBioscience, 13-0541-81, dilution-1:100), CD44-APC (eBioscience, 17-0441-81, dilution-1:100) and Streptavidin-PE-Cyanine7 (eBioscience, 25-4317-82, dilution-1:500) for 30 minutes, followed by two washing steps with FACS buffer (2% FCS in PBS). TNG-MKOS-MEFs were re-suspended in FACS buffer and analyzed on an LSRFortessa Cell Analyzer (BD Biosciences). Single-stained cells were used as controls. The data was analyzed using FlowJo software (FlowJo LLC).

For the time course mRNA-sequencing libraries, 3 to 15% TNG-MEFs were seeded and transduced with retroviral particles expressing transgenes. After inducing reprogramming, around 3x10^5^ mOrange positive cells were sorted on FACSAria IIIu cell sorter (BD Biosciences) and used the same for library preparation as described in [90].

## Additional files

Additional file 1: Gene set enrichment analysis of SRP016568. The file contains an XLSX spreadsheet summarizing the top 20 most enriched gene ontology (GO) terms among the 6278 genes that were significantly more highly expressed in iPSCs compared to foreskin fibroblasts in the study [21]. For comparison, the statistics for the same GO terms are also shown for the gene set enrichment analysis done using all 11705 differentially expressed genes. A total of 17249 genes were found to be expressed in the data sets and were used as background. The first four columns, from left to right, indicate the GO term identifier, name, ontology class/namespace, and the total number of (human) genes that are associated with the term. The next three columns indicate the number of genes associated with the term that were found more highly expressed in iPSCs, the false discovery rate (FDR; Benjamini-Hochberg) and the rank (by FDR) for the GO term enrichment. The remaining three columns indicate the same parameters for all differentially expressed genes. RNA-related GO terms have their names and identifiers in bold and italics. Asterisks next to GO term identifiers indicate that the term has the exact same set of genes associated with it as the preceding one and is thus redundant. (XLSX 6 kb)

Additional file 2: RNA-Seq sample table. The file contains an XLSX spreadsheet of the RNA-Seq data sets used in this study. For each sample listed are, from left to right, the Sequence Read Archive (SRA) study and run identifier, the organism and the cell type from which the sample was derived, and a descriptive sample group name that was used to pool samples for further analysis. (XLSX 14 kb)

Additional file 3: **Figure S1.** RNA-Seq library statistics. The following parameters were evaluated for all analyzed reprogramming endpoint RNA-Seq data sets and shown as bar-and-whisker plots, grouped by study: (A) number of reads, (B) read length, (C) percent mapped reads, (D) percent uniquely mapped reads. The Sequence Read Archive accessions for each study are indicated on the y axes. Medians are indicated as thick black horizontal lines. The lower and upper limits of boxes denote the first and third quartile, respectively, while whiskers indicate the 5th (bottom) and 95th (top) percentiles. Where applicable, outliers are indicated as circles. (PDF 17 kb)

Additional file 4: Table of comparisons for differential analyses. The file contains an XLSX spreadsheet describing sample groups that were used for differential and gene set enrichment analyses. Comparisons are always between the end and start points of reprogramming (end point / start point or, in log-space, end point - start point). The table lists, from left to right, the Sequence Read Archive (SRA) study identifier, the organism from which the samples were derived, the sample groups of reprogramming start and end points, and a short name linking the comparisons to figures. (XLSX 5 kb)

Additional file 5: **Figure S2.** Distribution of gene expression changes. (A) Absolute log2 fold changes in gene expression between all iPSC and all fibroblast samples, irrespective of the species and study, are depicted in a cumulative fraction plot. Only genes with exactly one ortholog in each of human, mouse and chimpanzee were considered. The data in red is from genes that are associated with GO term “RNA splicing” (GO:0008380), while the data in blue is from remaining genes. The statistic and *P* value of the Kolmogorov-Smirnov test calculated for the data sets is indicated. (B) As in (A), but log2 fold changes are depicted in density plots and statistics (Student’s *t*-test; *t* and corresponding *P* value) for the difference of the means are indicated. (C and D) as in (A and B), respectively, but data for genes associated (red) or not associated (blue) with GO term “RNA processing” (GO:0006396) is plotted. (E and F) as in (A and B), respectively, but data for genes associated (red) or not associated (blue) with GO term “gene expression” (GO:0010467) is plotted. (G and H) as in (A and B), respectively, but data for genes associated (red) or not associated (blue) with GO term “spliceosomal complex” (GO:0005681) is plotted. (I and J) as in (A and B), respectively, but data for genes associated (red) or not associated (blue) with GO term “ribosome” (GO:0005840) is plotted. (PDF 521 kb)

Additional file 6: **Figure S3**. Human reprogramming time course. The expression profile of splicing factors from Fig. 2a (y-axes, in TPM) shown as a function of time (in days; x axes), from the hiF-T reprogramming experiment (SRP049340) [28]. Dashed lines indicate 95% confidence intervals. (PDF 19 kb)

Additional file 7: **Figure S4.** Mouse reprogramming time course. As in Figure S3 but data is from mouse embryonic fibroblast reprogramming (study SRP059670) [15]. For each time point, data from single- (red) and paired-end (blue) RNA-Seq were available. (PDF 23 kb)

Additional file 8: **Figure S5**. Changes in splicing factor expression in cancers. Fold changes in expression of individual factors (from Fig. 2a) between cancers and corresponding healthy tissues are depicted. Data and tumor/cancer classifications are from The Cancer Genome Atlas (TCGA). Organisms and dendrograms as in Fig. 1d/e, splicing factor bins (orange, white, blue or mixed color boxes next to the gene symbols) as in Fig. 2a. (PDF 25 kb)

Additional file 9: **Figure S6.** RNA processing factors with consistent changes in gene expression. (A and B) As in Fig. 2a and b, respectively, but for genes (y axis; human gene symbol used) associated with GO term “RNA processing” (GO:0006396) and its children. Symbols of genes not associated with GO term “RNA splicing” (GO:0008380), which is a descendent of “RNA processing”, are highlighted (maroon). In contrast to Fig. 2a, splicing factors previously implicated in somatic cell reprogramming are only included if they exceeded the expression and fold change cut-offs. (PDF 82 kb)

Additional file 10: **Figure S7.** In vitro characterization of iPSCs. (A) qRT-PCR measurements of *Esrp1/2* expression 4 days after retroviral transduction of TNG-MKOS7-MEFs, relative to non-transduced TNG-MKOS-MEFs. Error bars indicate standard deviations (n = 3). See Additional file 16 for raw data. (B) Genomic PCR of iPSC clones demonstrating the integration of the indicated transgenes. M, 100 bp DNA marker (Invitrogen, 15628-019). (C) Relative expression of pluripotency markers in iPSC clones derived from TNG-MKOS-MEFs expressing the indicated transgenes compared to embryonic stem cells, as analyzed by qRT-PCR. ESC, embryonic stem cells (MKOS cassette) [87]. See Fig. 4d for abbreviations and Additional file 16 for raw data. (D) Germ layer specific marker expression analyzed by qRT-PCR after the induction of spontaneous differentiation in embryonic stem cells (ESC) and iPSC clones expressing the indicated transgenes. See Fig. 4d for abbreviations and Additional file 16 for raw data. (E) Images of embryoid bodies derived from iPSC clones carrying the indicated transgenes. Scale bar 100 μm. (F) Directed differentiation of iPSCs was performed as described previously [94]. Representative images of neurons derived from iPSC clones carrying the indicated transgenes. Green represents β-III tubulin (eBioscience, 14-4510-80) and blue represents nuclei stained with Hoechst dye. Scale bar: 10 μm. (PDF 537 kb)

Additional file 11: **Figure S8**. Analysis of alternative splicing. (A and B) As in Fig. 1a and b, but instead of gene expression values, the ‘percent spliced in’ (PSI) values calculated by SUPPA [76] for all indexed human (A) and mouse (B) alternative splicing events and then analyzed by principal component analysis. (C) Similar to Fig. 1d, but gene set enrichment analyses were performed for differentially spliced genes versus all genes with annotated isoforms. Log10 false discovery rates (FDR) of all GO terms (rows) that were found enriched (FDR < 0.1) in at least half of human (blue boxes above heatmap) or mouse (red boxes) comparisons (columns) are plotted. Highly significant values were capped at log10 FDR = -5, for clarity. GO term categories are indicated as an extra column to the right of the heatmap: BP, biological process; CC, cellular compartment; MF, molecular function. (PDF 43 kb)

Additional file 12: **Figure S9.** Splicing events in human and mouse reprogramming. The heatmaps depict ‘percent spliced in’ (PSI) values across the indicated comparisons (column labels) for the top 100 alternative splicing (AS) events (row labels), ranked by absolute mean z-scores across comparisons, in human (A) and mouse (B). Row labels represent shorthand event identifiers derived from gene symbols or, if unavailable, Ensembl gene identifiers. See Additional file 3 for the corresponding SUPPA event identifiers. AS event classes are indicated by colored boxes with the following abbreviations: A3, alternative 3’ splice site; A5, alternative 5’ splice site; AF, alternative first exons; AL, alternative last exon; MX, mutually exclusive exons; RI, retained intron; SE, skipping exon. Filtering of events by expression as in Fig. 5a. Dendrograms/clustering as in Fig. 1d/e. (PDF 62 kb)

Additional file 13: Top ranked alternative splicing events for human and mouse reprogramming. The file contains an XLSX spreadsheet with information on the 100 top ranked alternative splicing events derived from Ensembl gene annotations (release 84) [70], as identified in a set of endpoint-to-endpoint comparisons of human (first tab) or mouse (second tab) somatic cell reprogramming experiments. Splice sites were ranked according to mean z-scores. The unique shorthand event identifiers (Figure S9), as well as the corresponding SUPPA event identifiers, Ensembl gene identifiers, gene symbols and SUPPA event types are indicated. (XLSX 17 kb)

Additional file 14: **Figure S10.** Impact of Esrp1/2 overexpression on selected splicing events. TNG-MKOS-MEFs transduced with retroviruses harboring expression cassettes for either *Esrp1* (green), *Esrp2* (purple) or Luciferase (red), or not transduced (blue) were treated with doxycycline to induce reprogramming. RNA-Seq libraries (n = 1) were generated from samples taken at day 0 through 5 and day 15 (representing fully reprogrammed iPSCs). (A) Percent spliced in (PSI) values are plotted for each day for those of the mouse events in Additional file 3 that correspond to genes that have been associated with the network of splicing changes in Fig. 5c or have previously been identified as Esrp1/2 targets [15, 39]. (B) Total gene expression of *Esrp1* (top) and *Esrp2* (bottom) along the reprogramming time course. (C and D) The fractions of total *Grhl*1 gene expression (C) and abundances (D) of the *Grhl*1 isoforms 201 and 202 are indicated for each time point. (PDF 53 kb)

Additional file 15: Table of primers and PCR assays. The XLSX spreadsheet file contains a list of all primers used for the cloning of transgenes into retroviral vectors, qRT-PCR assays for pluripotency, differentiation, epithelial and mesenchymal markers, genomic PCR reactions for transgenes derived from iPSC clones, and *Cd44* RT-PCR reactions. (XLSX 7 kb)

Additional file 16: Raw data. Raw data values for relevant experiments are summarized in an XLSX spreadsheet. Data from each figure panel is presented in a single sheet. Labels correspond to those used in the figures. (XLSX 56 kb)

## Abbreviations

(c)iPSC: (chemically) induced pluripotent stem cells
(Δ)PSI: (delta) percent spliced in
APC: Adipose progenitor cells
EMT: Epithelial-mesenchymal transition
FB: Fibroblasts
GO: Gene ontology
hiF(-T): Human inducible fibroblast-like cells (constitutively express human Telomerase)
HPC: Hematopoietic progenitor cells
LB: Lymphoblastoid cells
MEF: Mouse embryonic fibroblasts
MET: Mesenchymal-epithelial transition
MKOS: Expression cassette harboring cDNAs for Myc, Klf4, Oct4 and Sox2
SSC: Spermatogonial stem cells
TNG-MKOS-MEF: Transgenic MEFs with MKOS cassette
TPM: Transcripts per million
