# Supplementary figures and images for: Conserved regulation of RNA processing in somatic cell reprogramming
Source: BMC Genomics. 2019 Jan 31;20:100. doi: 10.1186/s12864-019-5438-2 (PMC6357513; doi:10.1186/s12864-019-5438-2)

**A**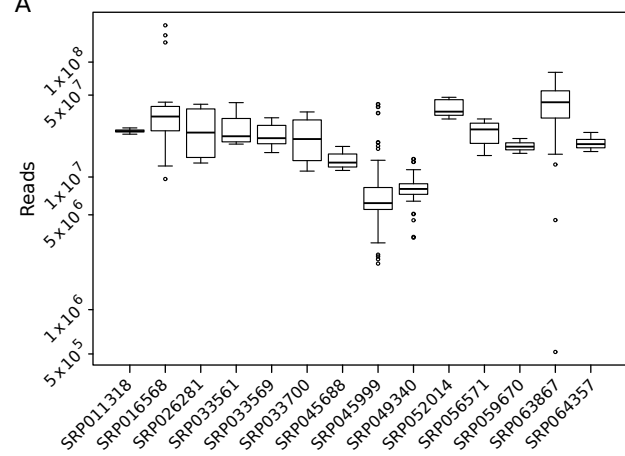**B**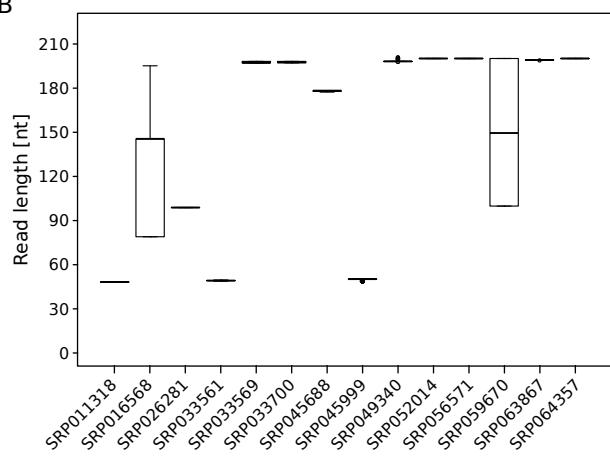**C**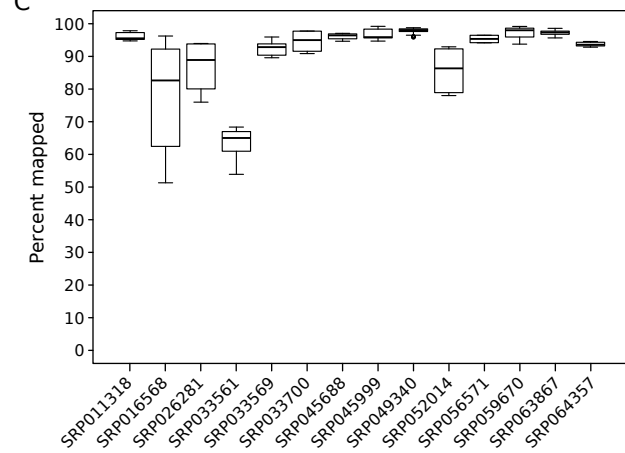**D**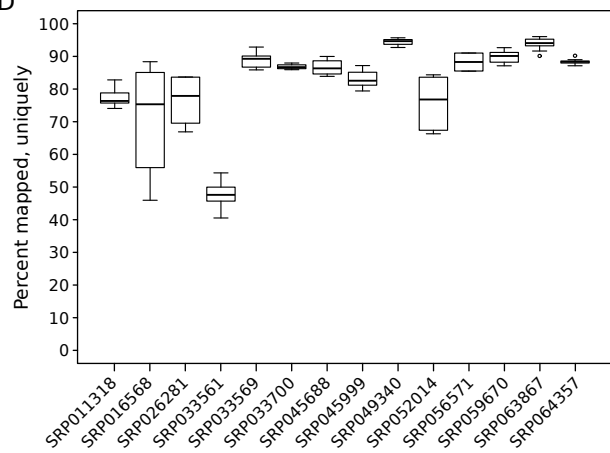

Supplement: Supplementary file 3 — Figure S1. RNA-Seq library statistics. The following parameters were evaluated for all analyzed reprogramming endpoint RNA-Seq data sets and shown as bar-and-whisker plots, grouped by study: (A) number of reads, (B) read length, (C) percent mapped reads, (D) percent uniquely mapped reads. The Sequence Read Archive accessions for each study are indicated on the y axes. Medians are indicated as thick black horizontal lines. The lower and upper limits of boxes denote the first and third quartile, respectively, while whiskers indicate the 5th (bottom) and 95th (top) percentiles. Where applicable, outliers are indicated as circles. (PDF 17 kb) [file 12864_2019_5438_MOESM3_ESM.pdf]

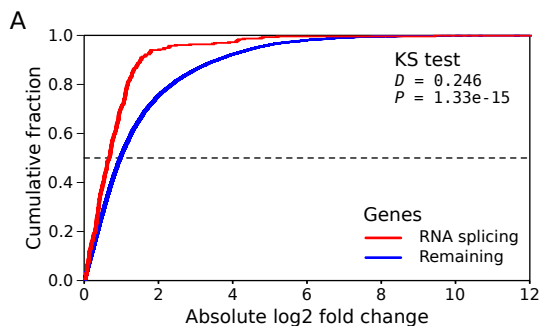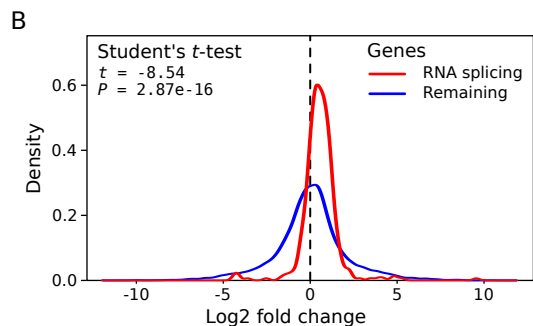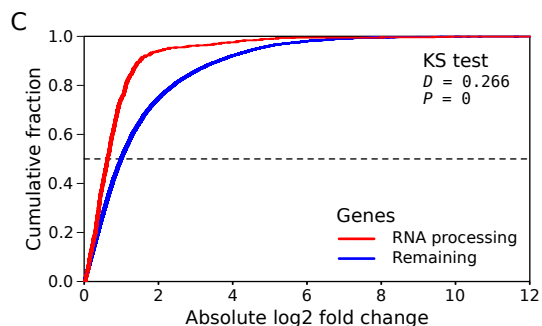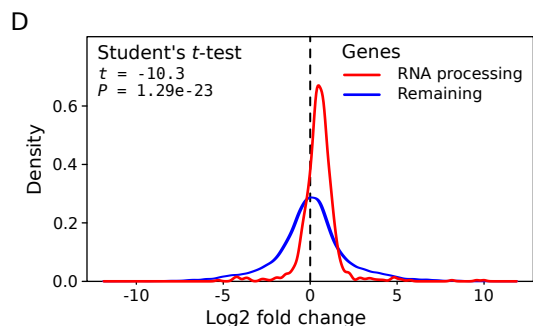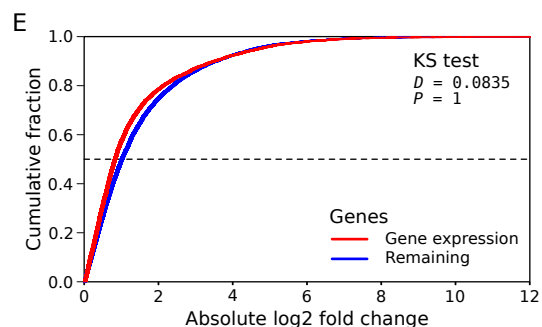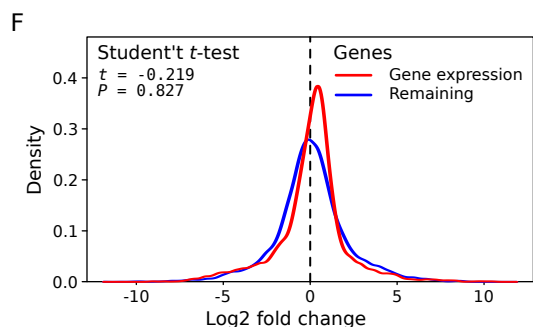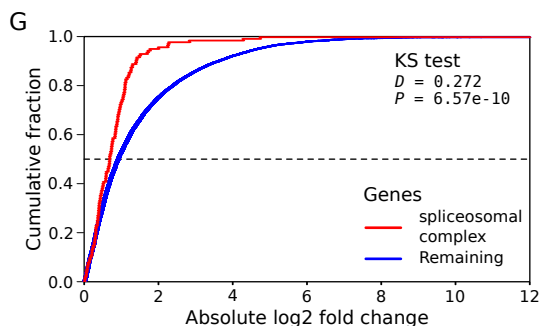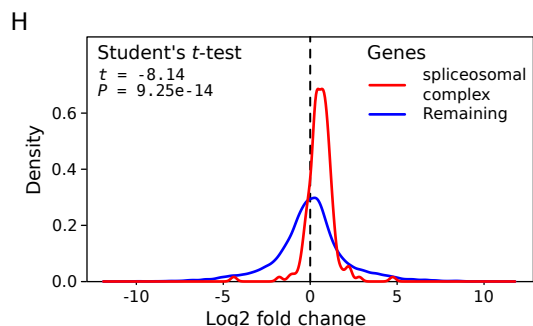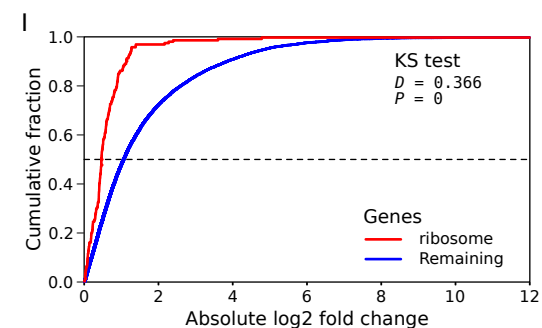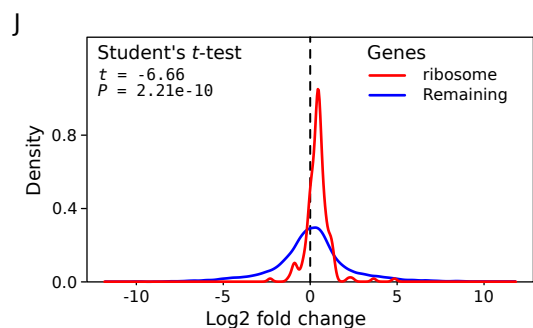

Supplement: Supplementary file 5 — Figure S2. Distribution of gene expression changes. (A) Absolute log2 fold changes in gene expression between all iPSC and all fibroblast samples, irrespective of the species and study, are depicted in a cumulative fraction plot. Only genes with exactly one ortholog in each of human, mouse and chimpanzee were considered. The data in red is from genes that are associated with GO term “RNA splicing” (GO:0008380), while the data in blue is from remaining genes. The statistic and P value of the Kolmogorov-Smirnov test calculated for the data sets is indicated. (B) As in (A), but log2 fold changes are depicted in density plots and statistics (Student’s t-test; t and corresponding P value) for the difference of the means are indicated. (C and D) as in (A and B), respectively, but data for genes associated (red) or not associated (blue) with GO term “RNA processing” (GO:0006396) is plotted. (E and F) as in (A and B), respectively, but data for genes associated (red) or not associated (blue) with GO term “gene expression” (GO:0010467) is plotted. (G and H) as in (A and B), respectively, but data for genes associated (red) or not associated (blue) with GO term “spliceosomal complex” (GO:0005681) is plotted. (I and J) as in (A and B), respectively, but data for genes associated (red) or not associated (blue) with GO term “ribosome” (GO:0005840) is plotted. (PDF 521 kb) [file 12864_2019_5438_MOESM5_ESM.pdf]

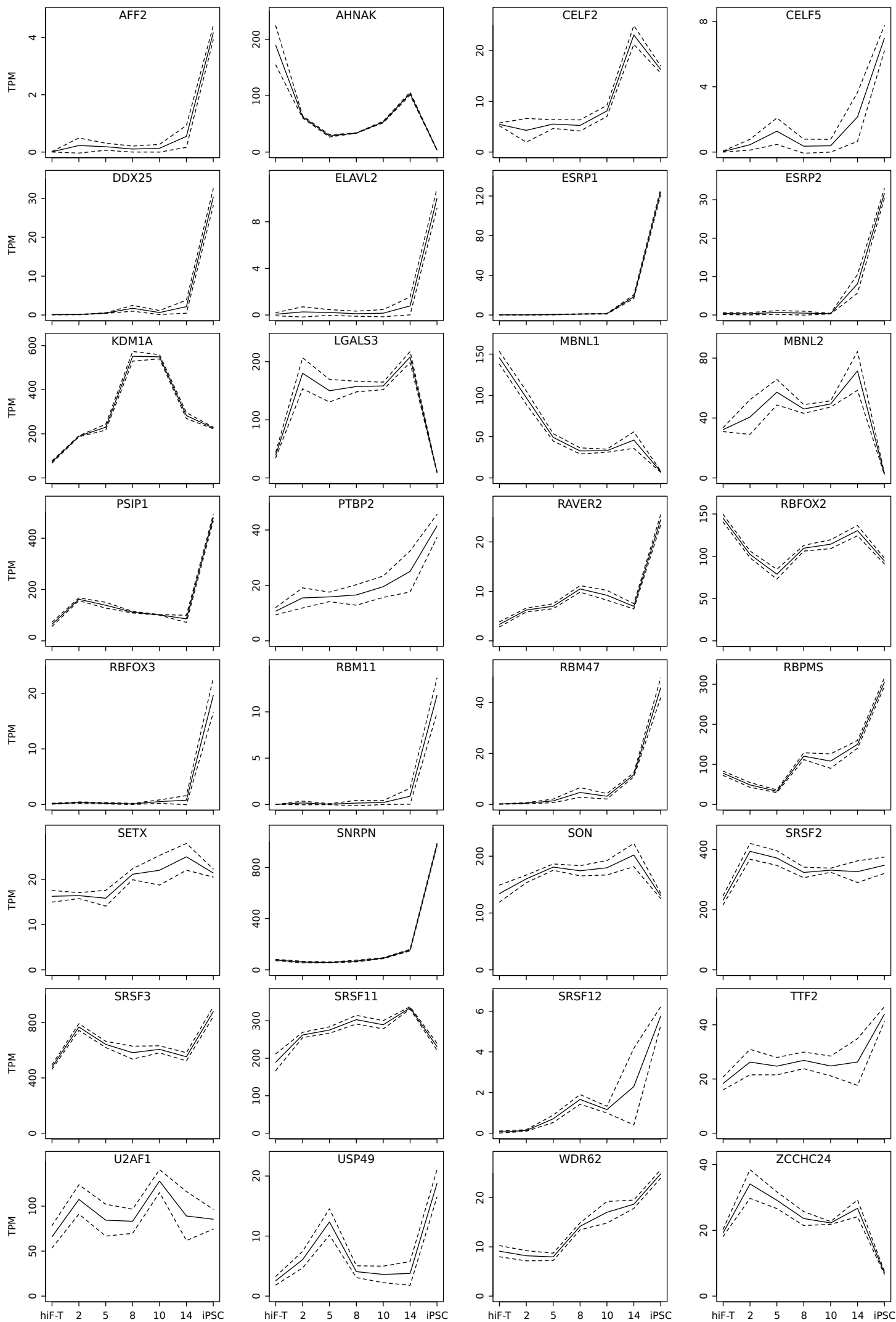

Supplement: Supplementary file 6 — Figure S3. Human reprogramming time course. The expression profile of splicing factors from Fig. 2a (y-axes, in TPM) shown as a function of time (in days; x axes), from the hiF-T reprogramming experiment (SRP049340) [28]. Dashed lines indicate 95% confidence intervals. (PDF 19 kb) [file 12864_2019_5438_MOESM6_ESM.pdf]

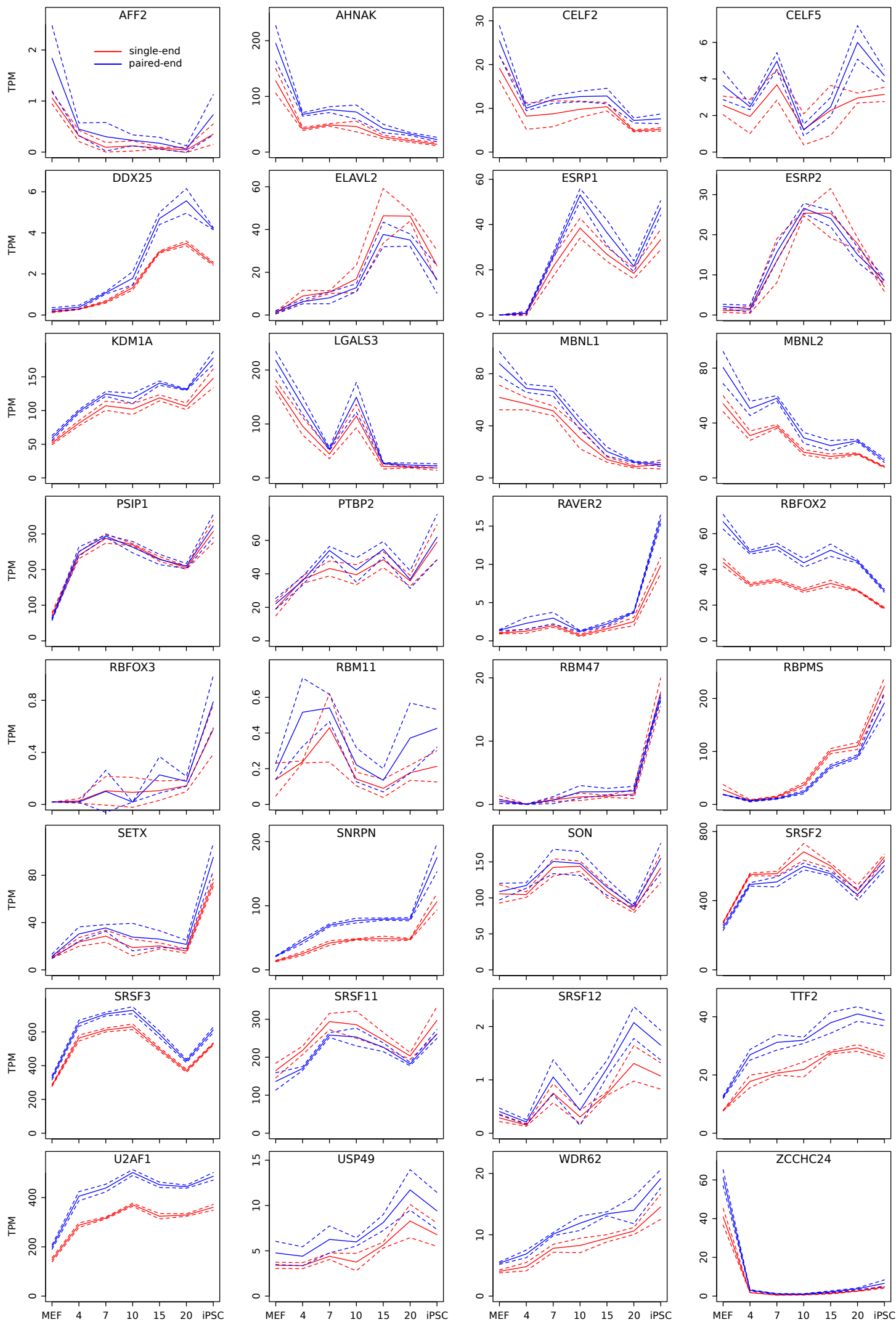

Supplement: Supplementary file 7 — Figure S4. Mouse reprogramming time course. As in Figure S3 but data is from mouse embryonic fibroblast reprogramming (study SRP059670) [15]. For each time point, data from single- (red) and paired-end (blue) RNA-Seq were available. (PDF 23 kb) [file 12864_2019_5438_MOESM7_ESM.pdf]

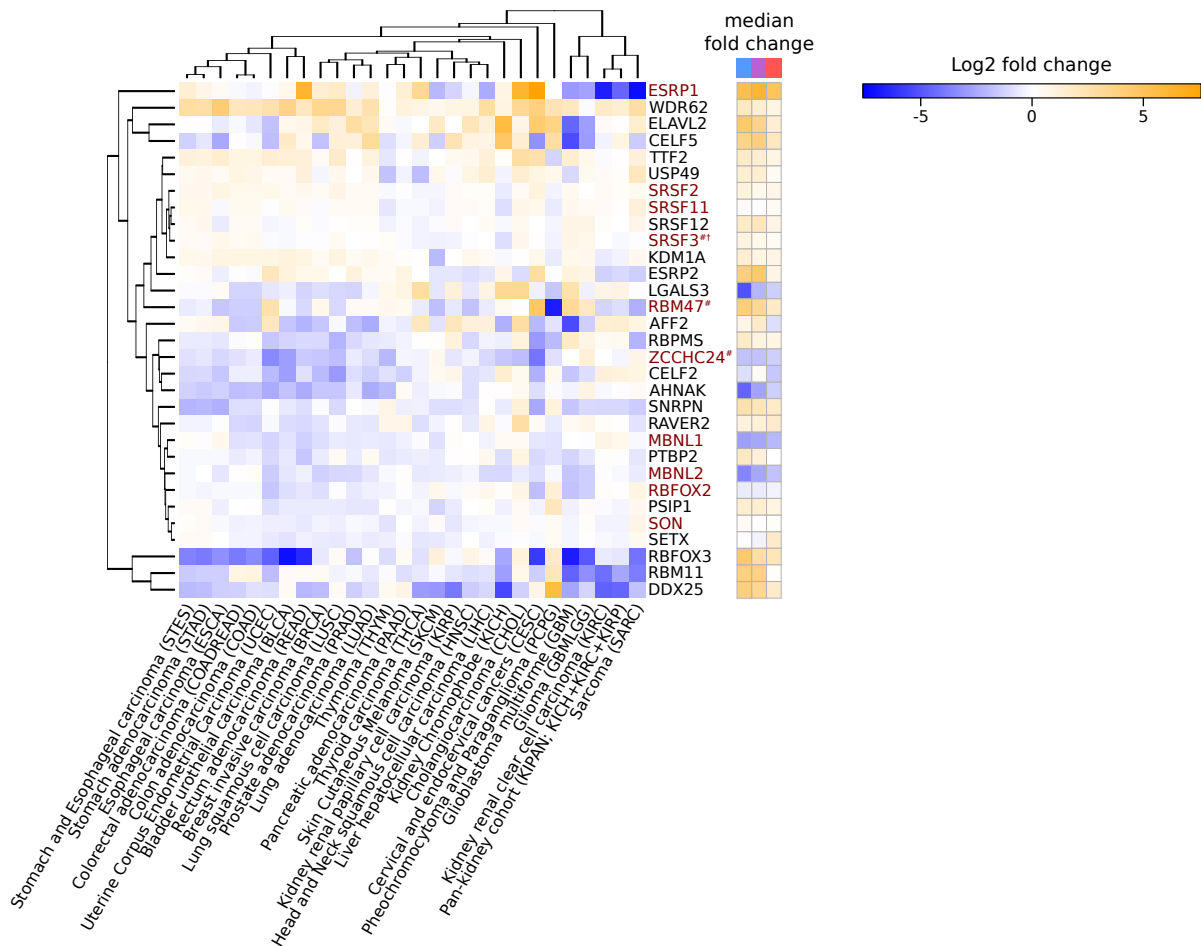

Supplement: Supplementary file 8 — Figure S5. Changes in splicing factor expression in cancers. Fold changes in expression of individual factors (from Fig. 2a) between cancers and corresponding healthy tissues are depicted. Data and tumor/cancer classifications are from The Cancer Genome Atlas (TCGA). Organisms and dendrograms as in Fig. 1d/e, splicing factor bins (orange, white, blue or mixed color boxes next to the gene symbols) as in Fig. 2a. (PDF 25 kb) [file 12864_2019_5438_MOESM8_ESM.pdf]

**A**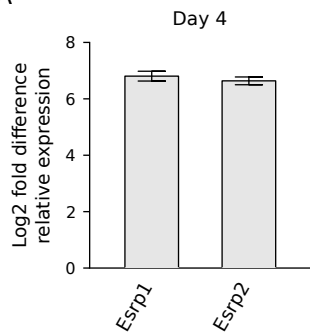**B**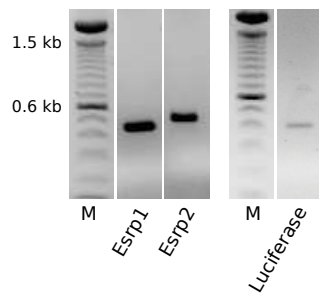**C**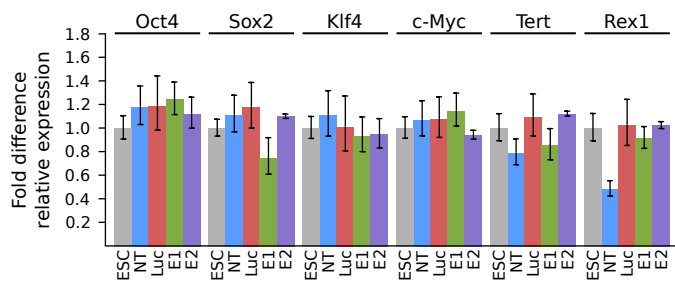**D**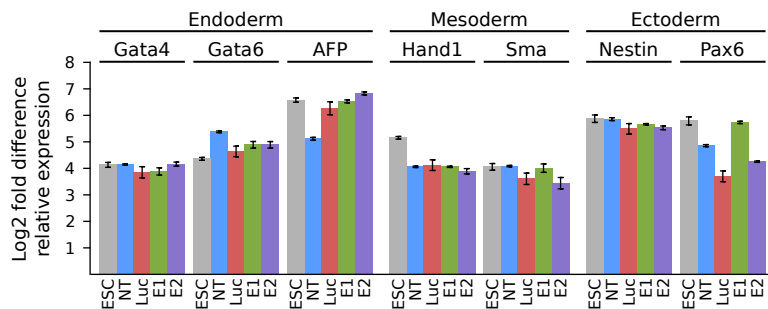**E**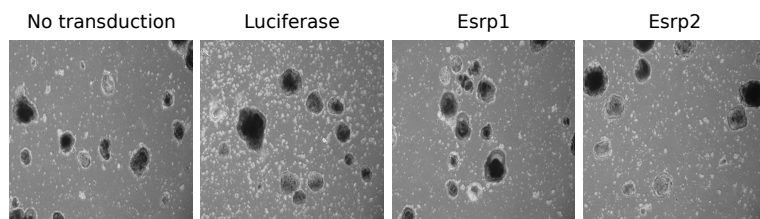**F**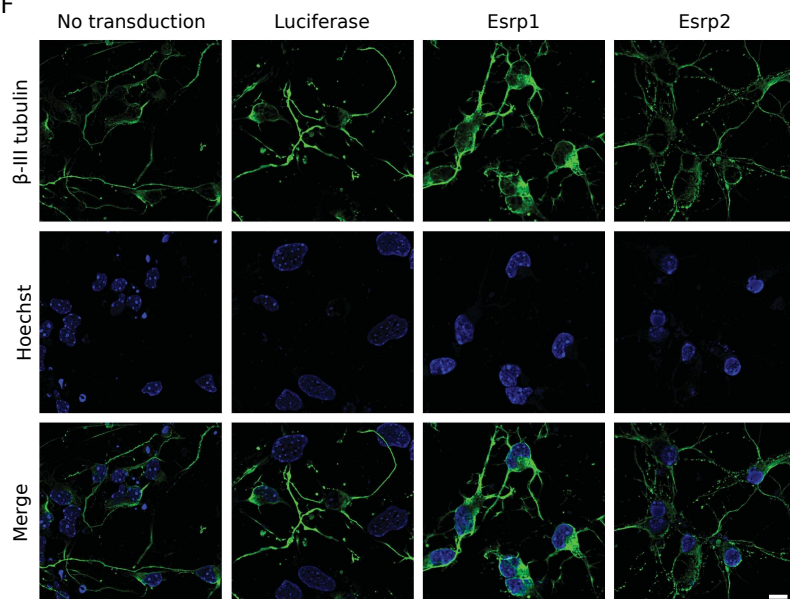

Supplement: Supplementary file 10 — Figure S7. In vitro characterization of iPSCs. (A) qRT-PCR measurements of Esrp1/2 expression 4 days after retroviral transduction of TNG-MKOS7-MEFs, relative to non-transduced TNG-MKOS-MEFs. Error bars indicate standard deviations (n = 3). See Additional file 16 for raw data. (B) Genomic PCR of iPSC clones demonstrating the integration of the indicated transgenes. M, 100 bp DNA marker (Invitrogen, 15628-019). (C) Relative expression of pluripotency markers in iPSC clones derived from TNG-MKOS-MEFs expressing the indicated transgenes compared to embryonic stem cells, as analyzed by qRT-PCR. ESC, embryonic stem cells (MKOS cassette) [87]. See Fig. 4d for abbreviations and Additional file 16 for raw data. (D) Germ layer specific marker expression analyzed by qRT-PCR after the induction of spontaneous differentiation in embryonic stem cells (ESC) and iPSC clones expressing the indicated transgenes. See Fig. 4d for abbreviations and Additional file 16 for raw data. (E) Images of embryoid bodies derived from iPSC clones carrying the indicated transgenes. Scale bar 100 μm. (F) Directed differentiation of iPSCs was performed as described previously [94]. Representative images of neurons derived from iPSC clones carrying the indicated transgenes. Green represents β-III tubulin (eBioscience, 14-4510-80) and blue represents nuclei stained with Hoechst dye. Scale bar: 10 μm. (PDF 537 kb) [file 12864_2019_5438_MOESM10_ESM.pdf]

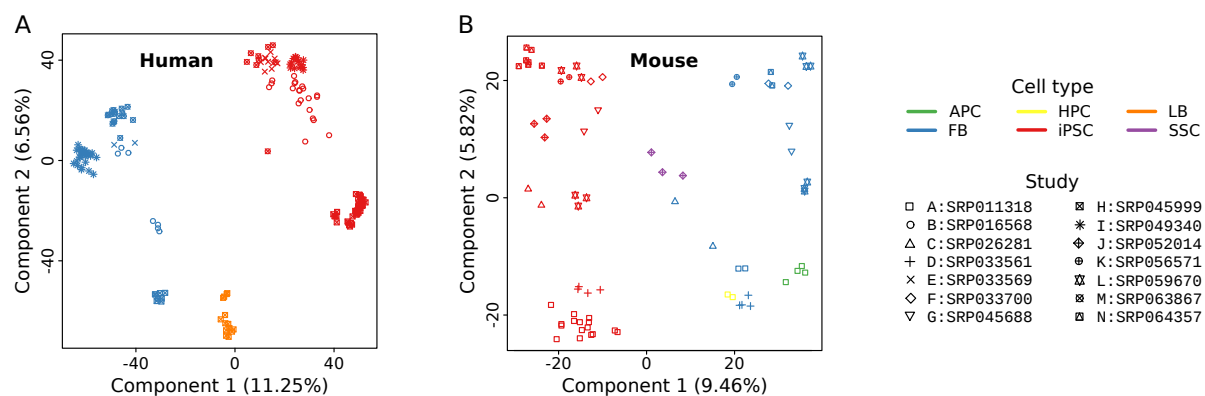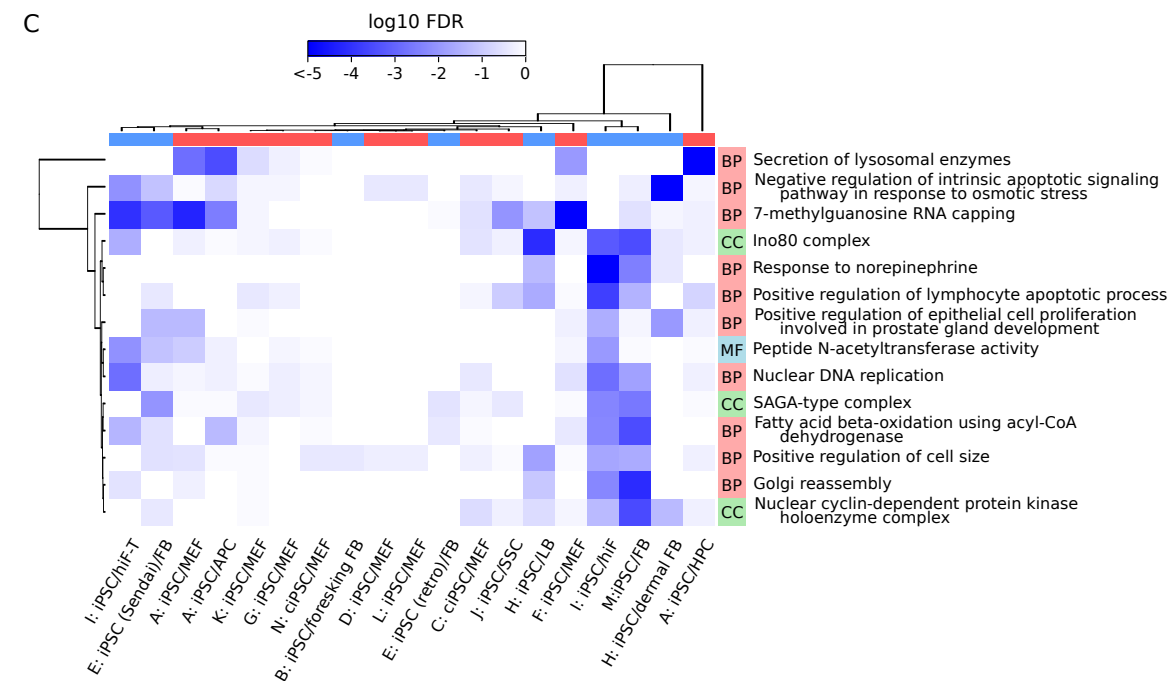

Supplement: Supplementary file 11 — Figure S8. Analysis of alternative splicing. (A and B) As in Fig. 1a and b, but instead of gene expression values, the ‘percent spliced in’ (PSI) values calculated by SUPPA [76] for all indexed human (A) and mouse (B) alternative splicing events and then analyzed by principal component analysis. (C) Similar to Fig. 1d, but gene set enrichment analyses were performed for differentially spliced genes versus all genes with annotated isoforms. Log10 false discovery rates (FDR) of all GO terms (rows) that were found enriched (FDR < 0.1) in at least half of human (blue boxes above heatmap) or mouse (red boxes) comparisons (columns) are plotted. Highly significant values were capped at log10 FDR = -5, for clarity. GO term categories are indicated as an extra column to the right of the heatmap: BP, biological process; CC, cellular compartment; MF, molecular function. (PDF 43 kb) [file 12864_2019_5438_MOESM11_ESM.pdf]

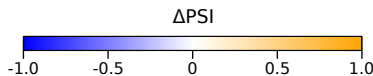

A

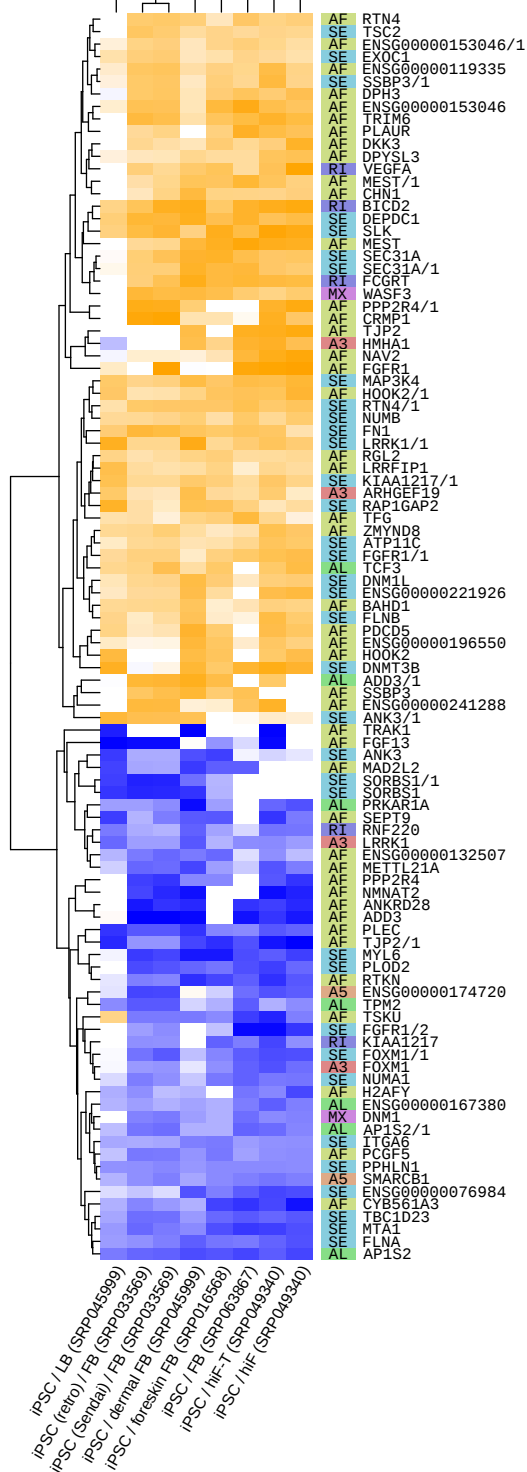

B

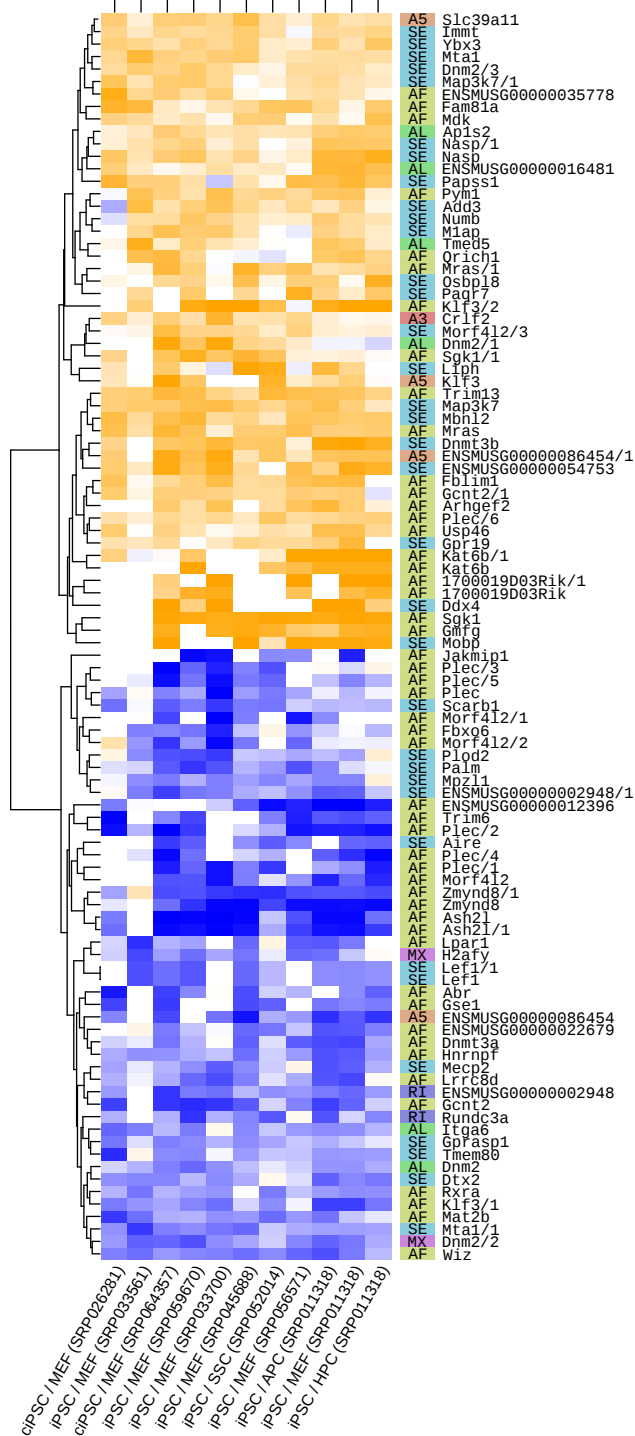

Supplement: Supplementary file 12 — Figure S9. Splicing events in human and mouse reprogramming. The heatmaps depict ‘percent spliced in’ (PSI) values across the indicated comparisons (column labels) for the top 100 alternative splicing (AS) events (row labels), ranked by absolute mean z-scores across comparisons, in human (A) and mouse (B). Row labels represent shorthand event identifiers derived from gene symbols or, if unavailable, Ensembl gene identifiers. See Additional file 3 for the corresponding SUPPA event identifiers. AS event classes are indicated by colored boxes with the following abbreviations: A3, alternative 3’ splice site; A5, alternative 5’ splice site; AF, alternative first exons; AL, alternative last exon; MX, mutually exclusive exons; RI, retained intron; SE, skipping exon. Filtering of events by expression as in Fig. 5a. Dendrograms/clustering as in Fig. 1d/e. (PDF 62 kb) [file 12864_2019_5438_MOESM12_ESM.pdf]

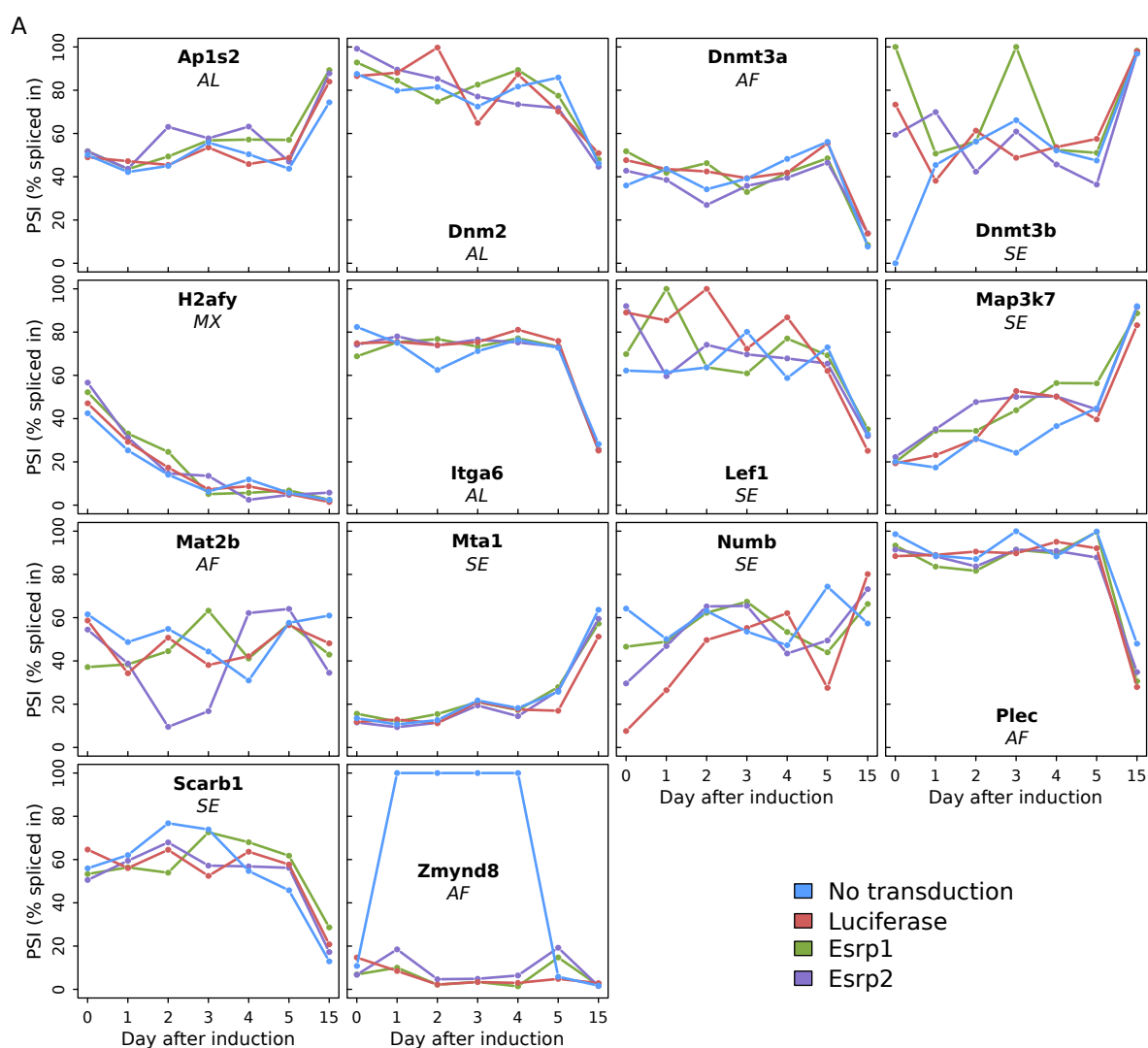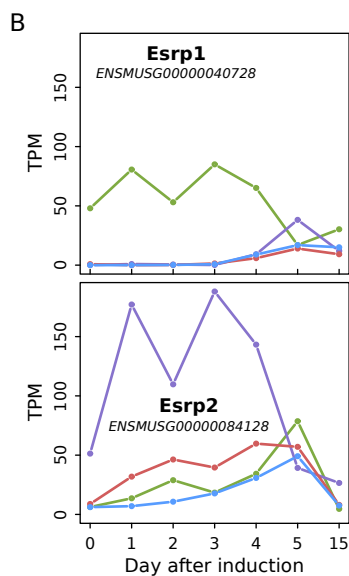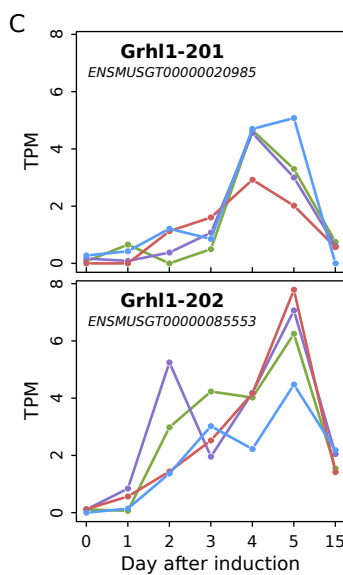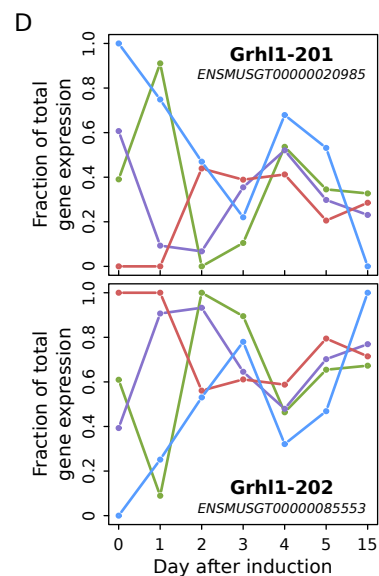

Supplement: Supplementary file 14 — Figure S10. Impact of Esrp1/2 overexpression on selected splicing events. TNG-MKOS-MEFs transduced with retroviruses harboring expression cassettes for either Esrp1 (green), Esrp2 (purple) or Luciferase (red), or not transduced (blue) were treated with doxycycline to induce reprogramming. RNA-Seq libraries (n = 1) were generated from samples taken at day 0 through 5 and day 15 (representing fully reprogrammed iPSCs). (A) Percent spliced in (PSI) values are plotted for each day for those of the mouse events in Additional file 3 that correspond to genes that have been associated with the network of splicing changes in Fig. 5c or have previously been identified as Esrp1/2 targets [15, 39]. (B) Total gene expression of Esrp1 (top) and Esrp2 (bottom) along the reprogramming time course. (C and D) The fractions of total Grhl1 gene expression (C) and abundances (D) of the Grhl1 isoforms 201 and 202 are indicated for each time point. (PDF 53 kb) [file 12864_2019_5438_MOESM14_ESM.pdf]
