# Supplementary material for: Conserved regulation of RNA processing in somatic cell reprogramming
Source: BMC Genomics. 2019 Jan 31;20:100. doi: 10.1186/s12864-019-5438-2 (PMC6357513; doi:10.1186/s12864-019-5438-2)
Supplement: Supplementary file 9 — Figure S6. RNA processing factors with consistent changes in gene expression. (A and B) As in Fig. 2a and b, respectively, but for genes (y axis; human gene symbol used) associated with GO term “RNA processing” (GO:0006396) and its children. Symbols of genes not associated with GO term “RNA splicing” (GO:0008380), which is a descendent of “RNA processing”, are highlighted (maroon). In contrast to Fig. 2a, splicing factors previously implicated in somatic cell reprogramming are only included if they exceeded the expression and fold change cut-offs. (PDF 82 kb) [file 12864_2019_5438_MOESM9_ESM.pdf]

A

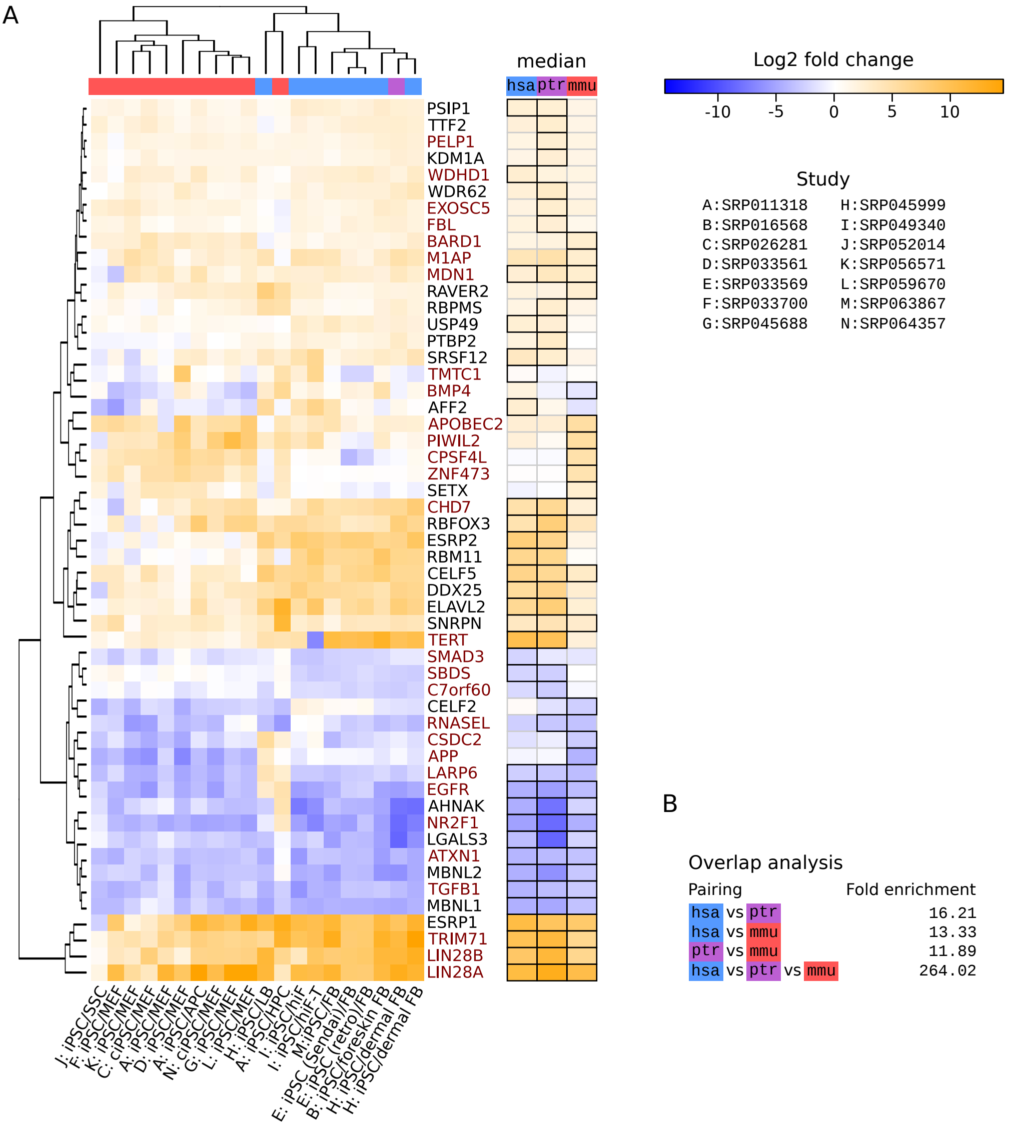

B

Overlap analysis

Pairing	Fold enrichment	*P* value
hsa vs ptr	16.21	2.75e-35
hsa vs mmu	13.33	1.49e-19
ptr vs mmu	11.89	2.38e-18
hsa vs ptr vs mmu	264.02	7.06e-45
